# Examining the effects of an eHealth intervention from infant age 6 to 12 months on child eating behaviors and maternal feeding practices one year after cessation: The Norwegian randomized controlled trial *Early Food for Future Health*

**DOI:** 10.1371/journal.pone.0220437

**Published:** 2019-08-23

**Authors:** Christine Helle, Elisabet R. Hillesund, Andrew K. Wills, Nina C. Øverby

**Affiliations:** 1 Department of Public Health, Sport and Nutrition, University of Agder, Kristiansand, Norway; 2 Faculty of Health Sciences, University of Bristol, Bristol, United Kingdom; Centre Hospitalier Universitaire Vaudois, FRANCE

## Abstract

**Objectives:**

The Norwegian randomized controlled trial *Early Food for Future Health* provided parental anticipatory guidance on early protective feeding practices from child age 6 to 12 months through an eHealth intervention. Previously published outcomes at child age 12 months indicated that the eHealth intervention increased daily vegetable/fruit intake and promoted more beneficial mealtime routines. The objective of the current paper is to evaluate the effects of the intervention at child age 24 months, one year after cessation.

**Methods:**

Parents of infants aged 3–5 months were recruited via social media and child health clinics during spring 2016. At child age 5.5 months, 715 mothers were randomized to either control (n = 358) or intervention (n = 360) arm. Primary study-outcomes were child eating behaviors, dietary intake, mealtime routines and maternal feeding practices and feeding styles. Secondary outcome was child anthropometry.

**Results:**

In total 295 mothers (41%) completed the follow-up questionnaire at child age 24 months. Regarding fruit intake, 54.3% in the intervention group had a high score compared with 48.3% of the control group (p = 0.29). For intake of vegetables, 54.5% in the intervention group had a high score compared with 50.7% in the control group (p = 0.49). A total of 65.7% of the children in the intervention group were eating breakfast together with family ≥ 4 times per week, compared with 57.3% of the children in the control group (p = 0.12). There was no difference between the groups for child anthropometric outcomes at child age 24 months.

**Conclusions:**

At child age 24 months, we found no evidence of sustained intervention-effects. Although dietary patterns and mealtime routines at child age 24 months were reasonably consistent and in the same directions as at child age 12 months, the between-group differences were not significant. The large loss to follow-up may have limited power and validity and makes it difficult to draw overall conclusions. Future research is needed to improve knowledge of how short-time effects could be retained over longer term, taking into account that larger samples are necessary when planning longer-term follow-up studies.

**Trial registration:**

ISRCTN, ISRCTN13601567.

## Introduction

The worldwide high prevalence of childhood overweight and obesity is a major public health concern in both developing and developed countries [1]. A large European prevalence study found that about 20% of children aged 2–10 years were overweight or obese [2]. A recent study from the US reported that 26% of preschool-aged children aged 2–5 years were overweight and 15.5% were obese or severely obese [3]. This early onset of childhood overweight and obesity underscores the need for obesity preventing strategies beginning in infancy. The first year of a child’s life constitutes a sensitive period for the establishment of biological determinants of risk trajectories, eating behaviors and dietary preferences. A substantial body of research suggests links between early infant nutrition and long-term development of non-communicable diseases (NCD) such as obesity, diabetes, hypertension and dyslipidemia [4–6]. The transition-phase from breast- or formula feeding to family foods therefore constitutes a temporal window where potential risk factors for later NCD can be prevented and healthy eating habits promoted.

During recent years, several early-life trials addressing modifiable parental- and child risk factors to prevent childhood overweight and obesity, have been developed [7–11]. Responsive feeding is characterized by caregiver guidance and recognition of the child’s cues of hunger and satiety [12]. Interventions using a responsive parenting framework have shown positive impacts on development of beneficial infant feeding practices [9], dietary patterns higher in fruits and vegetables [13], and reduced weight gain during infancy [14]. However, the effects of these interventions do not tend to be persistent across time [15–17]. Furthermore, the interventions have mostly been delivered using traditional approaches like home visits or face to face group settings, which is resource intensive and hence less able to be implemented at the level necessary for a national public health strategy. Despite the acknowledged importance of early-life prevention, there are still substantial gaps in our understanding of the most effective methods for promoting protective parental feeding practices [18, 19]. There is also a need for high-quality studies with longer-term follow-up to explore what is needed for effects to be maintained over time [19]. Web-based interventions have the potential to increase parental knowledge and skills while being both easily scalable and accessible. Research on the effectiveness of web-based behavioral change interventions is promising [20]. Yet, only very few studies have evaluated the effectiveness of interventions targeted at infant feeding practices and eating behaviors, and studies with longer term follow-up are warranted [21, 22].

The trial *Early Food for Future Health* started in 2015 with the objective of developing an eHealth intervention that targets beneficial parental feeding practices to promote healthy child eating behaviors from infancy. Details regarding study design and rationale for the intervention content have been previously published in a study protocol [23]. Outcomes at child age 12 months indicated that the eHealth intervention could increase daily vegetable/fruit intake and promote more beneficial mealtime routines [24]. The objective of the current paper is to evaluate the effects of the intervention one year after the intervention was ended, without further intervention components.

## Methods

### Study design

The study *Early Food for Future Health* is a primary prevention intervention, targeting parental feeding behaviors in the weaning period by offering anticipatory guidance on infant nutrition and protective feeding behavior through an eHealth intervention. The study uses the design of a randomized controlled trial (RCT) to evaluate the effects of the intervention, a completed CONSORT checklist can be found in Supporting Information, S1 Table. Measurements were taken using a web-based, self-administered questionnaire at baseline (child-age 5.5 months), at T1; after the intervention (child age 12 months) and at T2; one year after the intervention (child age 24 months). In the absence of response, up to two reminders were sent by email. The participants had the opportunity to not answer or complete the questionnaire without stating any reasons, hence we have no data regarding grounds for withdrawal. Outcomes at child age 12 months are previously reported [24], the outcome assessment at child age 24 months is reported here–se Supporting information S1 and S2 Files. The Norwegian Centre for Research Data evaluated and approved the study (http://pvo.nsd.no/prosjekt/43975), see Supporting Information S3–S5 Files.

### Recruitment, participants and randomization

Participants were recruited from March to June 2016. Inclusion criteria were having an infant age 3–5 months, being responsible for feeding the infant and being literate in Norwegian. Parents were recruited in two separate ways: By purchasing a Facebook advertising package including a short video that informed about the study, and by sending an email describing the study to all the Norwegian municipalities´ child health clinics. Informed consent from all participating parents were obtained upon registration. In total 960 parents signed up for participation on the study´s homepage http://spedbarnsmat.no/. Of these, 718 parents (715 mothers and 3 fathers) submitted the web-based baseline questionnaire at child age 5.5 months. Upon receipt of completed baseline questionnaire, the participants were individually randomized to control (N = 360) or intervention (N = 358) by the author based on a computer-generated list using the statistical software SPSS (IBM Corp., Somers; NY, USA). The participants were notified by email of group affiliation. Allocation was concealed from recruitment by virtue of the e-recruitment strategy.

### Treatment components

A webpage with a monthly age-appropriate video addressing infant feeding topics together with corresponding cooking films/recipes, were offered to participants in the intervention group from child age 6 to 12 months. The video clips were of 3–5 minutes duration and focused on feeding-related aspects like appropriate food-types and textures, development of taste-preferences, adequate and varied intake of fruits and vegetables and responsive feeding practices. The cooking-films demonstrated how to make homemade baby- and family food from healthy ingredients in an easy way. Parents in the control group received routine care from their local child health clinic with regular consultations at child age 6, 8, 10 and 12 months.

The eHealth intervention drew upon elements from attachment theory, social cognitive theory and the framework of anticipatory guidance to promote knowledge about infant nutrition and facilitate responsive feeding behavior. Video clips that addressed age-appropriate infant feeding topics and demonstrated sensitive parent-child interplay in feeding situations were used together with cooking films showing how to make healthy home-made baby food. The intervention was thought to improve parental feeding practices through social modelling and observational learning, and thereby lead to enhanced behavioral capability when it came to nutritional choices.

The participants' use and experience with the intervention were reported in our previous paper [24]. For the current paper, the participants compliance to the intervention was assessed by asking participants if they had seen the intervention`s infant feeding videos and cooking films *(all*, *most*, *about half*, *one or two*, *none)*.

### Outcomes and measurements

All socio-demographic and behavioral data were obtained from the study’s three web-based questionnaires: Baseline data at child age 5 months (T1) were collected between March and September 2016, data after termination of the intervention at child age 12 months (T2) between October 2016 and April 2017, and follow-up data at child age 24 months (T3) between October 2017 and April 2018. Our primary outcomes were child eating behaviors, dietary intake, mealtime routines and maternal feeding practices and feeding styles. Secondary outcome was child anthropometry.

#### Primary outcomes

Child eating behaviors were assessed using Wardle et al.´s *Child Eating Behavior Questionnaire* (CEBQ). This is a 35-item instrument with eight subscales developed for children above two years of age [25]. The eight subscales are divided into two main dimensions; The *Food approach dimension* and the *Food avoidance dimension*. The CEBQ has good internal consistency, test-retest reliability and construct validity [26], and has previously been translated to Norwegian [27]. For the Child Eating Behavior Questionnaire subscales, Cronbach´s α varied between 0.7 and 0.9, indicating a moderate to good internal consistency.

To evaluate the child´s willingness to try new food, we used the 6-item Child Food Neophobia Scale (CFNS) modified by Wardle et al. [28]. The CFNS is a reliable and widely used questionnaire which has been validated against behavioral measures of food neophobia [29]. A Norwegian version of CFNS has previously been translated and published [30]. The range of possible scores varies between 6 and 24, with a high score indicating high levels of child food neophobia. For the Child Food Neophobia Scale, Cronbach´s α was 0.9, indicating a good internal consistency.

Child food intake was assessed by a food frequency questionnaire developed for this study, based on questionnaires from a Norwegian national survey [31] and the large population-based Norwegian Mother and Child Cohort Study (MoBa) [32, 33]. The MoBa-study has documented their rationale for use of the FFQ [33], and a Norwegian validation study of the national survey FFQ has been done for child age 24 months [34]. Frequency of intake was assessed without specifications of amounts consumed. Questions about drink and food intake were asked as follows: *“How often does your child drink/eat the following beverages/food nowadays*?*”* with answer-options from *never/seldom* to ≥*5/day*. These responses were recoded into a times-per-day score, e.g. *1–3 times/week* was recoded into 0.3 times per day and *4–6 times/week* was recoded into 0.7 times per day.

We made four different food category scores by summing the individual scores in each category: In the *vegetable score* we included the times-per-day scores for 15 different vegetables *(potato*, *carrot*, *rutabaga*, *sweet potato*, *cauliflower*, *broccoli*, *green salad*, *spinach*, *cucumber*, *tomato*, *corn*, *peppers*, *peas/beans*, *frozen vegetable mix and salad of mixed raw vegetables)* and in the *fruit score* we included times-per-day scores for 10 different fruits/berries *(orange/clementine*, *banana*, *apple*, *pear*, *plum*, *grapes*, *kiwi*, *melon*, *mango*, *berries—all types)*. In the *sweet/salty-snack score*, we summed the times-per-week scores for five snacks *(cakes/cookies or similar*, *dessert/ice cream*, *chocolate*, *sweets*, *potato chips)* and in the *soft-drink score* we summed the times per week scores for sweetened beverages *(lemonade*, *soda)*. All four food-category scores were then dichotomized at the median intake into a *high* or *low* score.

Questions about mealtime routines were based on questions used in the Australian NOURISH-study [35]. Parents were asked “How often do the following statements fit the child's meals today?", e.g. *My child sits down when having meals*. Response-options were *almost always*, *often*, *sometimes*, *seldom*, *almost never*. These were subsequently recoded into always/often and sometimes/seldom/never. We further asked how often the child were eating family meals nowadays, specifying “family” to be at least one adult eating the same meal. Response options were *never/seldom*, *1 time/week*, *2 times/week*, *3 times/week*, *4 times/week*, *5 times/week*, *every day*. These were recoded into ≤ 3 times/week and ≥ 4 times/week.

The *Child Feeding Questionnaire* (CFQ) developed by Birch et.al. [36], is one of the most widely used questionnaires to assess parental feeding practices and examines parental attitudes and behaviors towards children´s diet. We included two subscales, *Restriction* and *Pressure*, to assess maternal controlling feeding practices in our sample. The CFQ has previously been translated to Norwegian and the same subscales were used in the Norwegian MoBa study [32]. Cronbach´s α for the *Restriction* and *Pressure* subscales of the Child Feeding Questionnaire was 0.66 and 0.63, respectively, indicating a moderate internal consistency for these subscales.

In addition, we used the *Parental Feeding Style Questionnaire* (PFSQ) designed by Wardle et al. [37], a tool for assessing four aspects of parental feeding styles; *Instrumental feeding*, *Encouragement*, *Emotional feeding* and *Control over eating*. PFSQ is a widely used questionnaire which shows good test-retest reliability and is validated in several cultures [38], and has also previously been used in Norway [27]. For the Parental Feeding Style Questionnaire subscales, Cronbach´s α varied between 0.7 and 0.8, indicating a moderate to good internal consistency.

#### Secondary outcomes

Child weight and length at baseline (5 months), 12, and 24 months were measured at the child health clinics and reported by the mothers. If the child had not been to the clinic at these time-points, mothers had the opportunity to omit responding. Z-scores for weight-for-age and body mass index (BMI)-for-age were calculated using the software program WHO Anthro version 3.2.2. (Department of Nutrition, World Health Organization, Geneva, Switzerland) and macros.

#### Sub-group analysis

At child age 12 months, we found no differences between mothers with lower and higher education for experiencing the intervention as feasible and easy to understand [24]. Since mothers with lower socioeconomic status constitutes a group of special interest in this field, we wanted to explore whether there were indications of any differences in the intervention’s suitability for this group compared to the entire study population. We performed a subgroup-analysis for the primary outcomes *child dietary intake* and *mealtime routines* including mothers who fulfilled at least one of the following criteria: 1) Lower education (*not college/university degree*); 2) Financial difficulties indicated by not being able to pay an unforeseen expense of 3000 NOK or having experienced difficulties paying their rent, food or transportation during the last six months; 3) Being a single mother (*not married/cohabitant*). The questions on financial difficulties were developed for the large population-based MoBa-study from the Norwegian Institute of Public Health to measure the family´s financial situation [33].

### Statistical analysis

Our sample size was decided based on the Australian NOURISH study, which used a similar population and assessed similar primary and secondary outcomes [35]. Considerations regarding sample size-calculations are previously published [23, 24]. It was estimated that we would need approximately 400 participants in both control and intervention arm, and we aimed to recruit 500 in each group to detect differences in feeding practices with 80% power and type 1 error of 5%, assuming a completion rate of 80%.

All analyses were conducted on an intention-to-treat basis, as the participants were analyzed as randomized irrespective of actual compliance to the intervention. Inferential statistics were used to test for potential between-group differences (intervention versus control) at baseline (Table 1). For the continuous variables *Child eating behavior*, *Child food neophobia* and *Maternal feeding style/-practices*, independent-samples t-test was used to test for between-group differences. The non-parametric test Mann-Whitney U test was used on the PFSQ sub-scale *Instrumental feeding* due to a non-normal distribution of scores on this subscale.

**Table 1:** Comparison of baseline characteristics between control and intervention group in the original sample and the sample retained in the study at child age 24 months.

Variable		Original sample at baseline Child age 5 months N _tot_ = 715			Sample retained at follow-up Child age 24 months N _tot_ = 343		
		Control (n = 358) % (count) or mean ± SD	Intervention (n = 357) % (count) or mean ± SD	P value	Control (n = 143–165)^1^ % (count) or mean ± SD	Intervention (n = 152–178)^1^ % (count) or mean ± SD	P value
**Mother**							
	Age (years)	30.2 ± 4.1	30.7 ± 4.5	0.10	30.5 ± 4.2	31.2 ± 4.4	0.13
	Not Norwegian as native language	7.8 (28)	7.3 (26)	0.77	9.7 (16)	5.6 (10)	0.16
	First-time mother (for infant participating in the survey)	57.7 (206)	56.1 (201)	0.67	59.4 (98)	59.3 (105)	0.99
	Marital status			0.20			0.68
	Married	36.4 (130)	42.7 (153)		37.6 (62)	41.8 (74)	
	Cohabitant	61.6 (220)	55.0 (197)		60.6 (100)	55.9 (99)	
	Not married/cohabitant	2.0 (7)	2.2 (8)		1.8 (3)	2.3 (4)	
	Education (College/university degree)	79.9 (283)	83.1 (295)	0.28	82.8 (135)	86.4 (152)	0.37
	Main activity			0.65			0.93
	Working fulltime	79.7 (283)	81.4 (289)		80.5 (132)	81.8 (144)	
	Working part time	7.3 (26)	6,2 (22)		6.7 (11)	5.7 (10)	
	Student	6.8 (24)	7.9 (28)		9.1 (15)	8.0 (14)	
	Not working	6.2 (22)	4.5 (16)		3.7 (6)	4.5 (8)	
	BMI (kg/m^2^)	24.8 ± 4.3	25.0 ± 4.4	0.51	24.5 ± 4.0	24.7 ± 4.3	0.69
	Smoking	3.6 (13)	3.9 (14)	0.85	3.0 (5)	2.8 (5)	0.91
	Use of snus	5.0 (18)	5.9 (21)	0.63	3.0 (5)	4.0 (7)	0.64
**Infant**							
	Gender (female)	48.7 (174)	49.7 (178)	0.79	50.3 (83)	48.6 (86)	0.75
	Gestational age>38 weeks	89.9 (321)	91.1 (326)	0.60	93.3 (154)	91.0 (161)	0.42
	Birth weight (g)	3587 ± 503	3573 ± 481	0.72	3580 ± 509	3570 ± 456	0.85
	Baseline weight (5 months)	7618 ± 910	7498 ± 912	0.13	7625 ± 899	7433 ± 793	0.076
	Exclusive breastfed first month	67.2 (240)	67.3 (241)	0.98	67.3 (111)	67.2 (119)	0.99
	Introduced to solid food before four months of age	4.8 (17)	4.7 (17)	0.99	4.8 (8)	4.5 (8)	0.89

^1^ The total number of participants in the analyses vary because of missing data for some variables

For the dichotomous variables *Child food intake* and *Selected mealtime routines*, odds ratios estimated with logistic regression were used as the measure of effect. For the child anthropometric outcomes BMI-for-age z-score and weight-for-age z-score, group means were compared using multiple linear regression, adjusting for the corresponding baseline values. A high number of child anthropometric values were missing at age 24 months, which may have led to biased estimates of the parameters. We therefore wanted to explore these data using imputations. Little's test was used to test for the assumption that data were missing completely at random. The expectation-maximization (EM) technique was used to estimate the missing values. Lastly, due to higher scores in the intervention group for the CEBQ-subscales *Emotional overeating* and *Food responsiveness*, which could potentially imply an adverse effect of the intervention in terms of increased weight gain, we explored the relationship between these subscales and BMI for age z-scores at 24 months using linear regression adjusting for group status and baseline BMI-for-age z-score. We further used logistic regression to explore the relationship between the *Food approach dimension* score and the dichotomous outcome child overweight and obesity. The data were analyzed by using IBM SPSS Statistics version 25.0 (IBM Corp., Somers; NY, USA).

## Results

### Characteristics of the study participants

**Fig 1:**
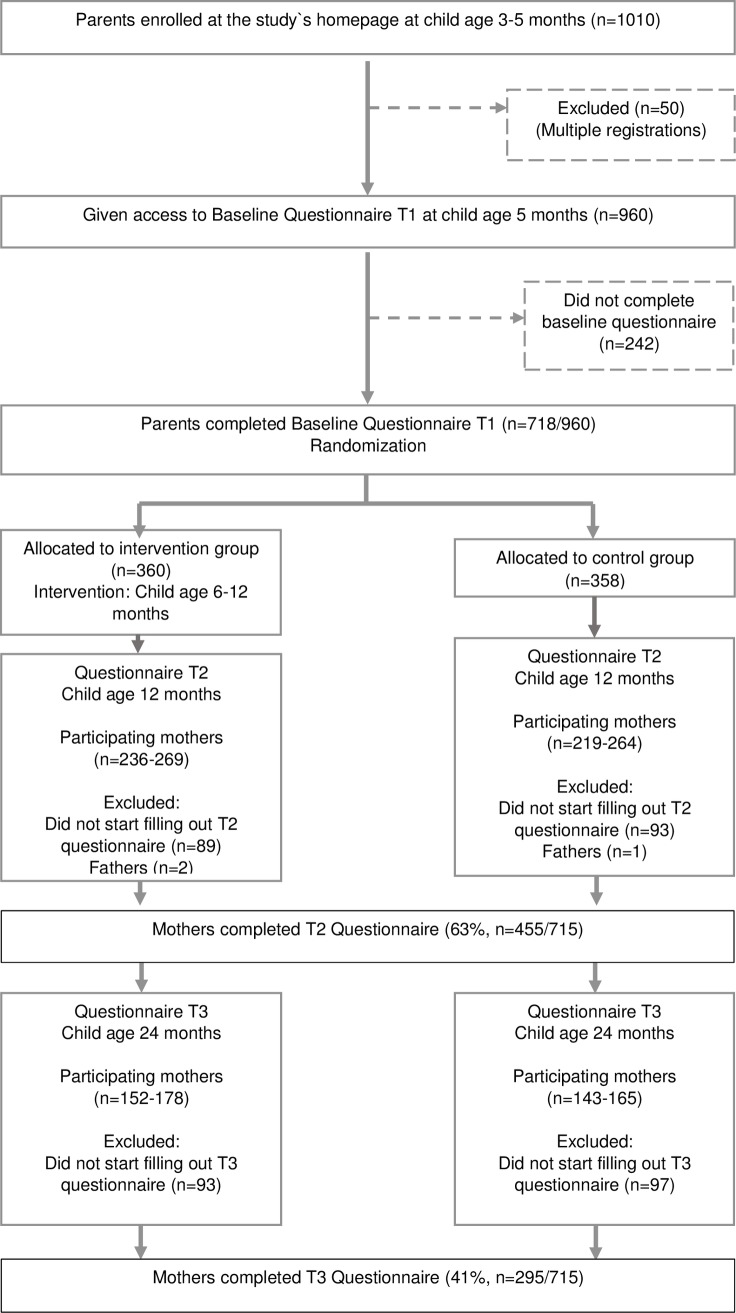
Flowchart of participants.

The flow of participants through the study is presented in Fig 1. From the 957 eligible participants recruited, 715 completed the baseline questionnaire at child age 5.5 months and were randomized to either control or intervention group. At 12 months of age, 455/715 (63%) mothers completed the follow-up questionnaire. Of these, 295 completed the follow-up questionnaire at child-age 24 months. The overall retention rate from allocation to follow-up at 24 months was 41% (295/715).

We compared baseline characteristics of mothers/infants who retained in the study with those lost to follow-up (Supporting Information, S2 Table). The mothers in the group lost to follow-up were on average slightly younger (30.1 years vs. 30.8 years; p = 0.02), and a slightly lower proportion had higher education (78.6% vs. 84.7%; p = 0.036). A higher proportion of the mothers in the lost-to-follow-up group used snus/powdered tobacco (7.3% vs. 3.5%; p = 0.027). No between-group differences were observed for the infants. Baseline maternal and child characteristics are presented by group status in Table 1. The groups were well balanced across the range of maternal and infant characteristics at baseline and follow-up suggesting no differential loss to follow-up between groups.

#### The participants´ compliance with the intervention

In the intervention-group, 81% of the mothers reported to have watched all or most of the videos addressing infant feeding topics, while 9% reported to have watched two or less. For the cooking videos, 73% of the mothers reported to have viewed all or most of the films while 10% had viewed two or less. This is quite consistent with what we found at child age 12 months, where 85% of the mothers reported watching all or most of the feeding videos and 66% reported watching all or most of the cooking films. The respective percentages for viewing less than two of the videos were 8% and 20% [24].

### Primary outcomes

#### Child eating behaviors

Table 2 shows the means and differences in means for the *Child eating behavior questionnaire* (CEBQ) and for *Child food neophobia* (CFNS). At child age 24 months we found a between-group difference for the subscales *Food responsiveness* and *Emotional over-eating*, with higher scores for both subscales in the intervention group. For willingness to try new food assessed by the *Child Food Neophobia Scale*, we found no difference between the groups.

**Table 2:** Child eating behavior, food neophobia and food-category intake at child age 24 months.

Item		Control mean ± SD (n = 143–167)^1^	Intervention mean ± SD (n = 152–176)^1^	Mean difference (95% CI)	P value
***Child Eating Behavior Questionnaire***^***2***^					
	Food responsiveness (5 items, α = 0.67)	2.16 ± 0.47	2.30 ± 0.61	0.14 (0.17 to 0.26)	0.026
	Emotional over-eating (4 items, α = 0.70)	1.48 ± 0.42	1.60 ± 0.48	0.12 (0.02 to 0.22)	0.019
	Enjoyment of food (4 items, α = 0.83)	3.89 ± 0.53	3.85 ± 0.55	-0.04 (-0.16 to 0.08)	0.49
	Desire to drink (3 items, α = 0.68)	2.71 ± 0.68	2.77 ± 0.68	0.06 (-0.09 to 0.22)	0.44
	Satiety responsiveness (5 items, α = 0.71)	3.05 ± 0.52	3.07 ± 0.51	0.02 (-0.10 to 0.13)	0.79
	Slowness in eating (4 items, α = 0.67)	2.72 ± 0.60	2.72 ± 0.63	-0.01 (-0.15 to 0.13)	0.91
	Emotional under-eating (4 items, α = 0.73)	3.17 ± 0.83	3.22 ± 0.73	0.05 (-0.13 to 0.22)	0.61
	Food fussiness (6 items, α = 0.90)	2.47 ± 0.77	2.43 ± 0.74	-0.05 (-0.22 to 0.12)	0.59
	CEBQ; Food approach dimension	2.56 ± 0.32	2.63 ± 0.37	0.07 (-0.01 to 0.15)	0.07
	CEBQ; Food avoidance dimension	2.85 ± 0.43	2.86 ± 0.41	0.01 (-0.09 to 0.10)	0.85
***Child Food Neophobia Scale*^*3*^*(Pliner)***					
	(6 items, α = 0.91)	12.01 ± 4.58	11.59 ± 4.49	-0.42 (-1.44 to 0.60)	0.42

Independent-samples t-tests are used for between-group comparison of continuous outcomes

^1^ The total number of participants in the analyses varies because of missing data for some variables

^2^ Behavior items rated from 1 (never) to 5 (always) on a five-point Likert scale. The mean sub-scale score is presented.

^3^ The range of possible responses varies between 6 and 24, with a high score indicating high levels of child food neophobia

#### Child dietary intake and mealtime routines

At child age 24 months, there was no evidence of a retained effect of the intervention on the combined fruits/vegetables score (6.51 ± 2.36 vs. 6.30 ± 2.26 for intervention versus control group; p = 0.41; data not shown). Table 3 presents child dietary intake of all the food-categories and mealtime routines at child age 24 months. We found no between-group differences for intake of fruits, vegetables, sweet and salty snacks or soft drinks. We neither found between-group differences for child eating mostly homemade versus commercially prepared dinner, child mealtime habits or frequency of family meals.

**Table 3:** Child dietary intake and mealtime routines at child age 24 months.

Item		Control % (count) (n = 143–167)^1^	Intervention % (count) (n = 152–176)^1^	OR^2^	CI 95%	P value
***Food category***, ***times per day/week***^***3***^						
	Fruits, ≥ 2.6 times per day	48.3 (73/151)	54.3 (89/164)	1.27	0.81–1.98	0.29
	Vegetables, ≥ 3.4 times per day	50.7 (77/152)	54.5 (90/165)	1.17	0.75–1.82	0.49
	Sweet and salty snacks, ≥ 3.5 times per week	68.6 (105/153)	61.2 (101/165)	0.72	0.54–1.15	0.17
	Soft drinks, ≥ 2.0 times per week	45.2 (70/155)	53.0 (88/166)	1.4	0.88–2.13	0.16
***Homemade / commercially prepared dinner***^***4***^						
	Child eating only/mostly homemade dinner	96.7 (146/151)	97.0 (159/164)	0.92	0.26–3.24	0.89
***Mealtime habits***^***5***^						
	Child eating the same for dinner as the rest of the family	98.1 (154/157)	96.4 (160/166)	0.52	0.13–2.11	0.36
	Child is sitting at the dinner table when eating	98.7 (155/157)	97.0 (161/166)	0.42	0.08–2.17	0.30
	Child playing or watching TV / tablet while eating	3.8 (6/157)	4.2 (7/166)	1.11	0.34–3.37	0.86
***Child eating meal together with family*^*6*^*≥ 4 times per week***						
	Breakfast	57.3 (90/157)	65.7 (111/169)	1.43	0.91–2.23	0.12
	Lunch	36.3 (57/157)	31.4 (53/169)	0.80	0.51–1.27	0.35
	Dinner	98.7 (155/157)	97.6 (165/169)	0.53	0.10–2.95	0.47
	Supper	45.9 (72/157)	51.5 (87/169)	1.25	0.81–1.94	0.31

Logistic regression is used for between-group comparison of dichotomous outcomes

^1^ The total number of participants in the analyses varies because of missing data for some variables

^2^ Control group as reference group

^3^ Answer-options were *never/not tried*, *‹ 1/week*, *1-2/week*, *3-4/week*, *5-6/week*, *1/day*, *2/day*, *3/day and ≥4/day* recoded into times-per-day and times-per-week scores. The scores were dichotomized by the median intake

^4^ Answer-options *Only homemade*, *Mostly homemade*, *Half of each*, *Mostly industrially made diner*, *Only industrially made dinner*

^5^ Answer options *Almost always*, *Often*, *Sometimes*, *Rarely* and *Almost never*. *Almost always/often* reported

^6^ *Family* defined as at least one adult eating the same meal

Subgroup analysis limited to the group of mothers with lower socio-economic status

In our sample 45 out of 293 (15.4%) of the mothers had a lower socioeconomic status defined by having either lower education, financial difficulties or being a single mother. The mean maternal age was the same as for the whole sample. The primary outcomes child dietary intake and mealtime routines for this sub-group are presented by allocation in Table 4. We found no significant differences between the groups, but the between-group differences were in the same directions as for the whole sample regarding intake of fruits and vegetables as well as for eating breakfast together with family.

**Table 4:** Child dietary intake and mealtime routines in the lower-SES group at child age 24 months.

Item		Control % (count) (n = 21)	Intervention % (count) (n = 24)	OR^1^	CI 95%	P value
***Food category***, ***times per day/week***^***2***^						
	Fruits, ≥ 2.6 times per day	42.9 (9)	50.0 (12)	1.33	0.41–4.33	0.63
	Vegetables, ≥ 3.4 times per day	47.6 (10)	62.5 (15)	1.83	0.56–6.03	0.32
	Sweet and salty snacks, ≥ 3.5 times per week	61.9 (13)	66.7 (16)	1.23	0.36–4.18	0.74
	Soft drinks, ≥ 2.0 times per week	42.9 (9)	58.3 (14)	1.87	0.57–6.11	0.30
***Child eating meal together with family***^***3***^ ***≥ 4 times per week***						
	Breakfast	42.9 (9)	58.3 (14)	1.87	0.57–6.11	0.30
	Lunch	42.9 (9)	37.5 (9)	0.80	0.24–2.64	0.72
	Dinner	95.2 (20)	95.8 (23)	1.15	0.07–19.60	0.92
	Supper	47.6 (10)	50.0 (12)	1.10	0.34–3.55	0.87

^1^ Control group as reference group

^2^ Answer-options were *never/not tried*, *‹ 1/week*, *1-2/week*, *3-4/week*, *5-6/week*, *1/day*, *2/day*, *3/day and ≥4/day* recoded into times-per-day and times-per-week scores. The scores were dichotomized at the median intake

^3^ *Family* defined as at least one adult eating the same meal

#### Maternal feeding practices/styles

Table 5 presents maternal feeding practices assessed by the *Child Feeding Questionnaire* and the *Parenting Feeding Style Questionnaire* at child age 24 months. We found no evidence for the intervention having an impact on maternal feeding practices or maternal feeding styles.

**Table 5:** Maternal feeding practices and feeding styles at child age 24 months.

Item		Control mean ± SD (n = 143–167)^1^	Intervention mean ± SD (n = 152–176)^1^	Mean difference (95% CI)	P value
***Child Feeding Questionnaire (CFQ)***^***2***^					
	Restriction (8 items, α = 0.66)	2.44 ± 0.60	2.49 ± 0.69	0.05 (-0.10 to 0.20)	0.52
	Pressure (4 items, α = 0.63)	2.11 ± 0.75	2.17 ± 0.74	0.06 (-0.11 to 0.23)	0.49
***Parental Feeding Style Questionnaire (PFSQ)***^***3***^					
	Instrumental feeding (4 items, α = 0.69)	1.39 ± 0.41	1.33 ± 0.45	-0.05 (-0.15 to 0.05)	0.09^4^
	Encouragement (8 items, α = 0.73)	3.99 ± 0.50	3.98 ± 0.45	-0.01 (-0.12 to 0.09)	0.80
	Emotional feeding (5 items, α = 0.80)	1.37 ± 0.42	1.35 ± 0.42	-0.02 (-0.12 to 0.08)	0.66
	Control over eating (10 items, α = 0.74)	4.02 ± 0.45	4.03 ± 0.40	0.01 (-0.09 to 0.10)	0.88

Independent-samples t-tests are used for between-group comparison of continuous outcomes

^1^ The total number of participants in the analyses varies because of missing data for some variables

^2^ PFSQ: 5-point likert-style scale (*1 = totally disagree to 5 = strongly agree)*. The mean sub-scale score is presented.

^3^ PFSQ: 5-point likert-style scale (*1 = never*, *2 = rarely*, *3 = sometimes*, *4 = often*, *5 = always)*. The mean sub-scale score is presented.

^4^ Between-group difference was assessed with the non-parametric test Mann-Whitney U test due to a non-normal distribution of scores on this sub-scale

### Secondary outcomes

#### Child anthropometric data

Table 6 shows the between-group differences in *weight-for-age* z-scores and *BMI-for-age* z-scores at child age 24 months. There was no difference between groups in *weight-for-age* z-score. For *BMI-for-age* z-score, we found a higher score in the intervention group. Descriptive growth-trajectories from birth to 24 months including all available data for the control and intervention group are presented in Supporting Information (S1 Fig).

**Table 6:** Difference in weight-for-age z-score and BMI-for-age WHO z-score at 24 months between groups (intervention minus control).

	Unadjusted (all available data)				Unadjusted complete cases			Adjusted for baseline values		
	n	Mean (SD)	Mean Difference (95% CI)	P value	n	Mean Difference (95% CI)	P value	n	Mean Difference (95% CI)	P value
**Weight-for-age z-score**										
**Intervention**	75	0.57 (0.90)	-0.04 (-0.35 to 0.28)	0.82	54	-0.03 (-0.41 to 0.34)	0.87	54	0.07 (-0.27 to 0.42)	0.67
**Control**	50	0.61 (0.81)			34			34		
**BMI-for age z-score**										
**Intervention**	72	0.63 (1.12)	0.10 (-0.29 to 0.49)	0.62	50	0.28 (-0.23 to 0.78)	0.28	50	0.53 (0.06 to 1.00)	0.029
**Control**	47	0.53 (0.95)			30			30		

Linear regression and multiple linear regression are used for between-group comparison of continuous outcomes

We used the expectation-maximization (EM) technique to estimate values for the missing child anthropometric values at age 24 months. When comparing the intervention and control group including imputed values, we found no significant between-group differences when adjusting for baseline values, neither for BMI-for-age z-score (mean difference 0.09 (-0.07 to 0.25); p = 0.29) nor weight-for-age z-score (mean difference -0.04 (-0.16 to 0.09); p = 0.55). However, these findings are only meant to be explorative and must be interpreted with caution.

#### Relationship between the *Child Eating Behavior Questionnaire* (CEBQ) subscales Food responsiveness and *Emotional over-eating* and child BMI

Due to higher scores in the intervention group for the CEBQ-subscales *Food responsiveness* (FR) and *Emotional over-eating* (EOE), we explored the relationships between each of these scores and *BMI-for-age* z-score at child age 24 months by multiple regression, adjusting for group status and baseline *BMI-for-age* z-score. We further explored the same relationship for the CEBQ composite-score *Food Approach Dimension*. Neither the subscales *Food responsiveness* (β = 0.36, p = 0.16), *Emotional over-eating* (β = 0.15, p = 0.60) nor the *Food approach dimension score* (β = 0.40, p = 0.30) were significant independent predictors of *BMI-for-age* z-scores at child age 24 months. Group allocation was also no independent predictor for child *BMI-for-age* z-score in any of the three models (β = 0.38 to 0.44, p = 0.12 to 0.18). Baseline *BMI-for-age* z-score was a strong independent predictor for *BMI-for-age* z-score at child age 24 months in all three models (β = 0.48 to 0.53, p = 0.002 to 0.005).

We further examined whether higher scores on the *Food approach dimension* was associated with the child being overweight/obese in our sample using logistic regression without adjusting for baseline values or group allocation. We used the revised, age-specific international IOTF BMI-cutoffs for overweight and obesity in children aged 2–18 years; child BMI ≥ 18.4 for boys and BMI ≥18.09 for girls aged 2 years [39]. In this sample, 12% (14/117) met the criteria for overweight or obesity. We found the *Food approach dimension* to be a significant independent predictor for overweight/obesity at child age 2 years (OR = 4.73; 95% CI = 1.0 to 22.40; *P* = 0.05).

## Discussion

### Principal findings of the study

The current paper reports outcomes of an eHealth intervention aiming to promote healthy food habits from infancy at child age 24 months, one year after the intervention was ended. Previously reported outcomes at child age 12 months provided promising evidence that the eHealth intervention may increase daily vegetable/fruit intake and promote more beneficial mealtime routines [24]. At child age 24 months, we found no evidence of effects of the intervention on these outcomes across time without further intervention. Still, there were similarities in between-group differences at 24 months of age compared with between-group differences shown at 12 months of age in our earlier report.

#### Child eating behavior, food-intake and mealtime routines

The sub-scale scores for the *Child Eating Behavior Questionnaire* in our sample were similar to previous scores for the same age group [40]. For the subscales *Food responsiveness* (FR) and *Emotional over-eating* (EOE) we found higher scores in the intervention group. These two subscales are included in the CEBQ composite-score *Food approach dimension*, which has been associated with increased food intake and overweight in children [41, 42], also as early as by the age of 2 years [43]. A higher score on this dimension could therefore be interpreted as an adverse finding. Our results at child age 24 months were similar to what we found at child age 12 months, where the intervention group had higher scores on the subscale *Food responsiveness* and the *Food approach dimension-*score [24]. When exploring the relationship between the *Food approach dimension*-score and *BMI-for-age* z-score at 12 months, adjusting for group status and baseline value of *BMI-for-age* z-score, we found that the *Food approach dimension score* was an independent positive predictor for *BMI-for-age* z-score at 12 months while group affiliation had no significant influence. At child age 24 months, neither *Food responsiveness*, *Emotional over-eating* nor the *Food approach dimension* significantly predicted *BMI-for-age* z-score at child age 24 months when adjusting for baseline *BMI-for-age* z-score and group status. Group allocation did not independently predict child *BMI-for-age* z-score at 24 months, while baseline *BMI-for-age* z-score was a strong positive independent predictor in all three models. Hence, the study’s intervention did not seem to have an adverse effect on the children’s weight-development. Nevertheless, for the whole sample we found the *Food approach dimension* to be associated with overweight/obesity at child age 2 years when not adjusting for baseline *BMI-for-age* z-scores. Our findings thereby support previous research indicating that eating behaviors measured by the CEBQ can be used to identify appetite-traits associated with weight status as early as by 2 years of age.

We found no impact of the intervention on child food neophobia measured by the *Child Food Neophobia Scale* (CFNS) at child age 24 months. Neither our earlier reported results at child age 12 months showed any between-group difference [24]. However, for both the control and intervention group the mean CFNS-scores were increased by 2.5, indicating an increased level of food neophobia at child age 24 months compared to child age 12 months. This is consistent with a normal eating development and in line with previous research finding that refusal of unfamiliar food begins in infancy, but peaks between 2 and 6 years of age [44].

A recently published Cochrane review on interventions for increasing fruit and vegetable consumption in children ≤ 5 years, concluded that the evidence for effective interventions remains sparse. Child feeding interventions appeared to increase fruit and vegetable intake, but this conclusion was based on very low-quality evidence, the effect sizes were small, and long-term follow-up was required [45]. This is consistent with findings from a recent review including 22 home-based and community-based interventions aiming to increase vegetable intake in children between 2 and 12 years [46]. Based on the calculated percentage change in vegetable intake, the included interventions reported a mean increase in vegetable intake of 29%, which equates to an increase of a quarter to a half of a serving of vegetables. Only ten of the studies reported long-term follow-up, of which six studies were considered to be effective also in longer term.

This study contributes to the existing body of research by demonstrating the small effect-sizes, the necessity of continuing support and the need for larger samples and longer-term follow-up. At child age 24 months, and without further intervention, we found no longer evidence for an impact of the intervention on child dietary intake of fruit, vegetables, sweet and salty snacks, soft drinks nor mealtime routines. Yet, the differences between the control and intervention group were still in the same direction as in our earlier reported outcomes at child age 12 months for intake of fruits and vegetables as well as for eating breakfast together with family [24]. When exploring potential differences in child food-intake and mealtime routines within the subgroup of mothers with lower socioeconomic status, we found the same directions for between-group differences, except for intake of sweet and salty snacks. Finding similar between-group differences for the control- and intervention group within the group of mothers with lower socioeconomic status, may support that the intervention did not differ in suitability depending on educational level or socioeconomic background.

#### Maternal feeding practices

Recognizing and responding appropriately to the infant’s cues of hunger and satiety is critical in supporting the infant’s innate capacity to self-regulate food-intake [47, 48]. Hence, the importance of a responsive feeding style was emphasized when designing the intervention. Despite this, we found no differences between the groups for maternal feeding practices or feeding styles neither at child age 12 nor 24 months. Whether the intervention’s web-based delivery mode is of importance remains unclear. Although previous studies using traditional intervention approaches have been able to show some impact on desirable parental feeding practices [15, 48, 49], the effects are small to modest and few long-term follow-up studies exist.

#### Child anthropometry

We found no impact of the intervention on child anthropometry at child age 12 months [24]. At child age 24 months, there was no between-group difference for weight-for-age z-score, but the intervention-group children had a higher BMI-for-age z-score. The meaning of these findings is unclear and the interpretation tentative, particularly due to missing values for more than half of the child anthropometric data. The sensitivity test using multiple imputations for missing data revealed no significant between-group differences. With reservations, we therefore interpret our findings in the sense that there is no strong evidence for a difference between the groups for child anthropometric outcomes at child age 24 months. This is consistent with previous research showing that although interventions may improve parental feeding practices or children’s nutrition and activity status, it is more difficult to demonstrate an impact on child growth. A systematic review including trials aiming to reduce the risk of overweight and obesity in infancy and early childhood, found that interventions addressing infant diet and parental responsiveness to infant cues, showed promise in terms of self-reported behavioral change but not weight [50]. Another systematic review including childhood obesity interventions in the first 1000 days, concluded that obesity interventions may have the greatest preventive effect if begun early in life, but so far few effective interventions exist [51]. Further, a 2014 meta-analysis of interventions aimed at reducing obesity in early childhood involving parents, concluded that although interventions may be effective at short-term follow-up, results are not retained in the long run [52]. The current study contributes to existing research by reaffirming the early onset of childhood overweight and obesity. In this sample consisting mostly of mothers with higher education, 12% of the children met the criteria for overweight or obesity at child age 2 years. Further, we found that growth trajectories associated with childhood overweight and obesity were established during infancy, as *BMI-for-age* z-score at 5 months was a strong positive independent predictor for *BMI-for-age* z-score at child age 24 months.

### Unanswered questions and future research

Even though early-life interventions targeting modifiable parental and child risk factors associated with childhood overweight and obesity hold significant promise, there are still substantial gaps in the knowledge of how short-time effects may be retained over longer term. A recent review of early-life obesity prevention trials concluded that the design of interventions needs to be considered within a life-course framework, requiring frequent interventions addressing multiple factors at various stages of life and have longer-term childhood outcomes [53]. The current study provides additional support to these conclusions, finding that effects on dietary intake and mealtime routines at child age 12 months were not maintained one year later without further intervention. Also, important considerations should be taken when planning new research. When effect sizes in trials aiming to increase child intake of fruit and vegetables seems to be consistently small [46], and the same accounts for weight-related outcomes [51], future research needs to consider the need for much larger samples when planning longer-term follow-up studies.

### Strengths and limitations

The study’s strengths include the randomized controlled design and a participant group including both first-time mothers and mothers with older children. As it is typically more difficult to change existing behaviors than to establish new ones, our findings are likely to be conservative. The public health utility of the intervention is important to recognize. The current study has generated new knowledge about short- and long-term effects of an early-life, web-based intervention with potential to be easily scaled-up and used at community level.

A major limitation is the lack of power for several of the between-group comparisons. Despite a comparatively large number of participants recruited, the retention rate over time was lower than foreseen. A total of 1010 participants registered themselves on the study´s homepage, but the actual number turned out to be 960 because of multiple registrations. In addition, more participants than expected dropped out between registration and baseline and between baseline and each of the follow-up time-points at child age 12 and 24 months. In Norway, maternity leave usually ends when the child is one year old, and most mothers then return to work. Busier work days with a lack of time can probably explain the high attrition between 12 and 24 months. The loss to follow-up may have influenced study results by limiting power and validity. However, the dropouts appear to be balanced between the intervention and the control group, so a mechanism for introducing bias is difficult to conceive. The mothers in the present study volunteered for participating, which in epidemiological research is known to lead to a more well-educated study-sample [54, 55] as seen in our study. The use of self-reported data may reduce reliability, and the nature of the sample may have implications for the generalizability of our findings.

## Conclusion

In our previously reported outcomes of the *Early Food for Future Health* study at child age 12 months, we found promising evidence that an eHealth intervention providing parental anticipatory guidance on early protective feeding practices from child age 6 to 12 months could increase daily vegetable/fruit intake and promote more beneficial mealtime routines. At child age 24 months, we found no lasting effects of the intervention without further continuing support. Although dietary patterns and mealtime routines at child age 24 months seemed to be reasonable consistent and to a large extent in the same directions as at child age 12 months, the between-group differences lacked precision due to a higher loss to follow-up than expected. The large loss to follow-up may have limited power and validity and makes it difficult to interpret findings and draw overall conclusions. Still, even small reductions in early-life risk factors may be of importance when carried through into later childhood and beyond. Further research is needed to improve knowledge of how short-time beneficial effects may be retained over longer term and how these strategies can be adopted and implemented to the magnitude needed for sustainable childhood obesity prevention.

## Supporting information

S1 Table CONSORT checklist. (PDF)

S2 Table Group comparisons of baseline characteristics. Comparison between participants who retained in the study and those lost to follow-up.
(PDF)

S1 File Study questionnaire at follow-up (child age 24 months), Norwegian version. (PDF)

S2 File Study questionnaire at follow-up (child age 24 months), English version. (PDF)

S3 File Norwegian regional committee for medical and health research; decision. (PDF)

S4 File The Norwegian Centre for Research Data; application and approval. (PDF)

S5 File The Norwegian Centre for Research Data; change notification and approval. (PDF)

S1 Fig Mean Weight-for-age z-score and mean BMI z-score for the control and intervention groups from birth to 24 months of age including all available data. (PDF)
