# Supplementary material for: Examining the effects of an eHealth intervention from infant age 6 to 12 months on child eating behaviors and maternal feeding practices one year after cessation: The Norwegian randomized controlled trial Early Food for Future Health
Source: PLoS One. 2019 Aug 23;14(8):e0220437. doi: 10.1371/journal.pone.0220437 (PMC6707582; doi:10.1371/journal.pone.0220437)
Supplement: S2 Table — Comparison between participants who retained in the study and those lost to follow-up. (PDF) [file pone.0220437.s002.pdf]

S2 Table:

Baseline characteristics of mothers/infants lost to follow-up compared with the participants who retained in the study

**Variable**	**Lost to follow-up (baseline-24 months) N=372**	**Retained in the study (study sample) at 24 months N=343**	**P value**
**Mother**			
Age	30.1 ± 4.3	30.8 ± 4.3	**0.020**
Not Norwegian as native language	7.5 (28)	7.6 (26)	0.98
First-time mother (for infant participating in the survey)	54.8 (204)	59.2 (203)	0.24
Marital status			0.98
Married	39.2 (146)	39.9 (137)	
Cohabitant	58.6 (218)	58.0 (199)	
Not married/cohabitant	2.2 (8)	2.0 (7)	
Education (College/university degree)	78.6 (290)	84.7 (288)	**0.036**
Main activity			0.32
Working fulltime	79.9 (295)	81.2 (277)	
Working part time	7.3 (27)	6,2 (21)	
Student	6.2 (23)	8.5 (29)	
Not working	6.5 (24)	4.1 (14)	
BMI (kg/m^2)	25.2 ± 4.5	24.6 ± 4.3	0.11
Smoking	4.6 (17)	2.9 (10)	0.25
Use of snus	7.3 (27)	3.5 (12)	**0.027**
**Infant**			
Gender (female)	48.9 (182)	49.6 (170)	0.87
Gestational age>38 weeks	89.2 (332)	91.8 (315)	0.24
Birth weight (g)	3585 ± 502	3575 ± 482	0.78
Weight baseline (5 months)	7585 ± 968	7576 ± 847	0.923
Exclusive breastfed first month	72.8 (101)	69.1 (106)	0.27
Introduced to solid food before four months of age	4.8 (18)	4.7 (16)	0.91
