# Supplementary material for: Examining the effects of an eHealth intervention from infant age 6 to 12 months on child eating behaviors and maternal feeding practices one year after cessation: The Norwegian randomized controlled trial Early Food for Future Health
Source: PLoS One. 2019 Aug 23;14(8):e0220437. doi: 10.1371/journal.pone.0220437 (PMC6707582; doi:10.1371/journal.pone.0220437)
Supplement: S1 File — (PDF) [file pone.0220437.s003.pdf]

Tusen takk for at du vil være med videre i barnE-mat studien!

Det enkleste er å fylle ut spørreskjemaet ved en dataskjerm. Du navigerer i skjemaet ved å trykke på pilene / "neste-knappen" nederst på siden.

Etter noen innledende spørsmål er spørreskjemaet todelt:
Første del av spørreskjemaet handler om barnet som deltar i undersøkelsen. Du vil trenge barnets helsekort for opplysninger om vekt og høyde, ha gjerne dette klart før du begynner. Andre del er om deg som er mor eller far til barnet.

Lykke til!

Hva er din relasjon til barnet som deltar i undersøkelsen?

(1) ❑ Jeg er mor

(2) ❑ Jeg er far

(3) ❑ Jeg er ingen av delene, beskriv _____

Hva er barnets fødselsdato?
Må skrives år-mnd-dag, f.eks. 2015-06-26

______

Hva er barnets kjønn?

(1) ❑ jente

(2) ❑ gutt

Er barnet som deltar i denne studien ditt eneste barn?

(0) ❑ Ja

(1) ❑ Nei

Hvor mange barn har du?

(1) ❑ To

(2) ❑ Tre

(3) ❑ Fire eller fler

Var du i gruppen som hadde tilgang til nettstedet og filmene i barnE-mat? (intervensjonsgruppen)

(1) ❑ Ja
(2) ❑ Nei

Så du de månedlige tema-filmene?

(1) ❑ Ja, så alle filmene
(2) ❑ Ja, så de fleste filmene
(3) ❑ Ja, så cirka halvparten av filmene
(4) ❑ Ja, så en eller to av filmene
(5) ❑ Nei, så ingen av filmene

Så du på mat-filmene eller oppskriftene?

(1) ❑ Ja, så alle filmene
(2) ❑ Ja, så de fleste filmene
(3) ❑ Ja, så cirka halvparten av filmene
(4) ❑ Ja, så en eller to av filmene
(5) ❑ Nei, så ingen av filmene

Nå følger noen spørsmål om barnets vekst og utvikling det siste året.

Ta utgangspunkt i barnets helsekort fra helsestasjonen og fyll inn dato for undersøkelse, vekt og lengde i spørsmålene. Har du ikke aktuelle vekt- eller høydemål, kan du gå videre i skjemaet.

Undersøkelse ved ca. 18 mnd. alder:

Dato for undersøkelse _____

Vekt i gram _____

Lengde i cm _____

Undersøkelse ved ca. to års alder:

Dato for undersøkelse _____

Vekt i gram _____

Lengde i cm _____

Nå følger noen spørsmål om barnets kosthold og matvaner ved to års-alder:

Hvor ofte pleier barnet ditt å spise følgende måltider i løpet av en uke?

	Aldri/sjelden	1 gang/uke	2 g/u	3 g/u	4 g/u	5 g/u	6 g/u	Hver dag
Frokost	(1) ❑	(2) ❑	(3) ❑	(4) ❑	(5) ❑	(6) ❑	(7) ❑	(8) ❑
Formiddagsmat/lunsj	(1) ❑	(2) ❑	(3) ❑	(4) ❑	(5) ❑	(6) ❑	(7) ❑	(8) ❑
Mellommåltid før middag	(1) ❑	(2) ❑	(3) ❑	(4) ❑	(5) ❑	(6) ❑	(7) ❑	(8) ❑
Middag	(1) ❑	(2) ❑	(3) ❑	(4) ❑	(5) ❑	(6) ❑	(7) ❑	(8) ❑
Mellommåltid etter middag	(1) ❑	(2) ❑	(3) ❑	(4) ❑	(5) ❑	(6) ❑	(7) ❑	(8) ❑
Kveldsmat	(1) ❑	(2) ❑	(3) ❑	(4) ❑	(5) ❑	(6) ❑	(7) ❑	(8) ❑
Andre måltider/mellommåltider	(1) ❑	(2) ❑	(3) ❑	(4) ❑	(5) ❑	(6) ❑	(7) ❑	(8) ❑

Hvor ofte spiser barnet følgende måltider sammen med familien nå?
Dvs. samtidig som en voksen spiser samme måltid

	Aldri/sjelden	1-3 ganger per uke	4-6 ganger per uke	Hver dag
Frokost	(1) ❑	(2) ❑	(3) ❑	(4) ❑
Formiddagsmat/lunsj	(1) ❑	(2) ❑	(3) ❑	(4) ❑
Middag	(1) ❑	(2) ❑	(3) ❑	(4) ❑
Kveldsmat	(1) ❑	(2) ❑	(3) ❑	(4) ❑

Er det du som oftest gir barnet mat?

(1) ❑ Ja
(2) ❑ Nei
(3) ❑ Deler likt

Pleier barnet å bli matet (dvs. en voksen holder skjeen eller deler opp maten og gir den bit for bit), eller spiser det selv?

	Spiser alltid selv	Spiser som oftest selv	Både og	Blir som oftest matet	Blir alltid matet
Frokost	(1) ❑	(3) ❑	(4) ❑	(5) ❑	(2) ❑
Lunsj	(1) ❑	(3) ❑	(4) ❑	(5) ❑	(2) ❑
Ettermiddagsmat (måltid etter lunsj og før middag)	(1) ❑	(3) ❑	(4) ❑	(5) ❑	(2) ❑
Middag	(1) ❑	(3) ❑	(4) ❑	(5) ❑	(2) ❑
Kveldsmat	(1) ❑	(3) ❑	(4) ❑	(5) ❑	(2) ❑
Andre måltider/mellommåltider	(1) ❑	(3) ❑	(4) ❑	(5) ❑	(2) ❑

Hvor ofte stemmer utsagnene under på barnets måltider nå?

	Nesten alltid	Ofte	Av og til	Sjelden	Nesten aldri
Barnet spiser samme middagsmat som resten av familien	(1) ❑	(6) ❑	(7) ❑	(8) ❑	(9) ❑
Jeg/vi lager egen middagsmat til barnet	(1) ❑	(6) ❑	(7) ❑	(8) ❑	(9) ❑
Barnet sitter ved bordet når hun/han spiser	(1) ❑	(6) ❑	(7) ❑	(8) ❑	(9) ❑
Barnet leker eller ser på TV/nettbrett/smarttelefon mens hun/han spiser	(1) ❑	(6) ❑	(7) ❑	(8) ❑	(9) ❑

Har eller har barnet hatt problemer med spising/mat?
Her kan du sette flere kryss

(1) ❑ Nei, har ingen problemer

(2) ❑ Ja, dårlig matlyst/småspist

(3) ❑ Ja, liker få matvarer

(4) ❑ Ja, vanskelig med tilvenning til familiens kosthold

(5) ❑ Ja, allergi/intoleranse mot enkelte matvarer

(6) ❑ Ja, andre problemer - beskriv _____

Hvor ofte får barnet følgende å drikke nå for tiden?

	Aldri/sjelden	1-3 ganger per uke	4-6 ganger per uke	1 gang per døgn	2 ganger per døgn	3 ganger per døgn	4 ganger per døgn	5 eller flere ganger per døgn
Vanlig søt melk, alle typer	(1) ❑	(2) ❑	(3) ❑	(4) ❑	(5) ❑	(6) ❑	(7) ❑	(8) ❑
Surmelk, alle typer (yoghurt, kulturmelk o.l.)	(1) ❑	(2) ❑	(3) ❑	(4) ❑	(5) ❑	(6) ❑	(7) ❑	(8) ❑
Sjokolademelk, alle typer	(1) ❑	(2) ❑	(3) ❑	(4) ❑	(5) ❑	(6) ❑	(7) ❑	(8) ❑
Vann	(1) ❑	(2) ❑	(3) ❑	(4) ❑	(5) ❑	(6) ❑	(7) ❑	(8) ❑

Hvor ofte får barnet følgende å drikke nå for tiden?

	Aldri/sjelden	1-3 ganger per uke	4-6 ganger per uke	1 gang per døgn	2 ganger per døgn	3 ganger per døgn	4 ganger per døgn	5 eller flere ganger per døgn
Saft, sukret	(1) ❑	(2) ❑	(3) ❑	(4) ❑	(5) ❑	(6) ❑	(7) ❑	(8) ❑
Saft, kunstig søtet	(1) ❑	(2) ❑	(3) ❑	(4) ❑	(5) ❑	(6) ❑	(7) ❑	(8) ❑
Juice	(1) ❑	(2) ❑	(3) ❑	(4) ❑	(5) ❑	(6) ❑	(7) ❑	(8) ❑
Brus, sukret	(1) ❑	(2) ❑	(3) ❑	(4) ❑	(5) ❑	(6) ❑	(7) ❑	(8) ❑
Lettbrus, kunstig søtet	(1) ❑	(2) ❑	(3) ❑	(4) ❑	(5) ❑	(6) ❑	(7) ❑	(8) ❑

Hvor ofte spiser barnet følgende mat nå for tiden?

Hvor ofte?

	Aldri/sjelden	1-3 g/u	4-6 g/u	1 g/døgn	2 g/døgn	3 g/døgn	4 el.flere g/døgn
Industrifremstilt grøt, alle typer	(1) ❑	(2) ❑	(3) ❑	(4) ❑	(5) ❑	(6) ❑	(7) ❑

Hvor ofte?

	Aldri/sjelden	1-3 g/u	4-6 g/u	1 g/døgn	2 g/døgn	3 g/døgn	4 el.flere g/døgn
Hjemmelaget grøt av grovt/sammalt mel, hirse eller havregryn/havremel	(1) ❑	(2) ❑	(3) ❑	(4) ❑	(5) ❑	(6) ❑	(7) ❑
Hjemmelaget grøt av fint/hvitt mel, kavring, semule, ris, eller mais	(1) ❑	(2) ❑	(3) ❑	(4) ❑	(5) ❑	(6) ❑	(7) ❑

Hvor ofte spiser barnet følgende mat nå for tiden?

	Aldri/ikke forsøkt	Under 1 g/uke	1-2 g/uke	3-4 g/uke	5-6 g/uke	1 g/døgn	2 g/døgn	3 g/døgn	4 g eller mer/døgn
Brød med leverpostei	(1) ❑	(8) ❑	(2) ❑	(3) ❑	(9) ❑	(4) ❑	(5) ❑	(6) ❑	(7) ❑
Brød med annet kjøttpålegg	(1) ❑	(8) ❑	(2) ❑	(3) ❑	(9) ❑	(4) ❑	(5) ❑	(6) ❑	(7) ❑
Brød med fiskepålegg	(1) ❑	(8) ❑	(2) ❑	(3) ❑	(9) ❑	(4) ❑	(5) ❑	(6) ❑	(7) ❑
Brød med ost	(1) ❑	(8) ❑	(2) ❑	(3) ❑	(9) ❑	(4) ❑	(5) ❑	(6) ❑	(7) ❑
Brød med syltetøy/honning	(1) ❑	(8) ❑	(2) ❑	(3) ❑	(9) ❑	(4) ❑	(5) ❑	(6) ❑	(7) ❑
Brød med annet pålegg	(1) ❑	(8) ❑	(2) ❑	(3) ❑	(9) ❑	(4) ❑	(5) ❑	(6) ❑	(7) ❑

Hvor ofte spiser barnet følgende mat nå for tiden?

	Aldri/ikke forsøkt	Under 1 g/uke	1-2 g/uke	3-4 g/uke	5-6 g/uke	1 g/døgn	2 g/døgn	3 g/døgn	4 g eller mer/døgn
Kjøtt, kjøttdeig, kjøttboller, pølse o.l.	(1) ❑	(8) ❑	(2) ❑	(3) ❑	(9) ❑	(4) ❑	(5) ❑	(6) ❑	(7) ❑
Fisk, fiskeboller-/kaker/-pudding o.l.	(1) ❑	(8) ❑	(2) ❑	(3) ❑	(9) ❑	(4) ❑	(5) ❑	(6) ❑	(7) ❑
Pannekaker	(1) ❑	(8) ❑	(2) ❑	(3) ❑	(9) ❑	(4) ❑	(5) ❑	(6) ❑	(7) ❑
Pizza	(1) ❑	(8) ❑	(2) ❑	(3) ❑	(9) ❑	(4) ❑	(5) ❑	(6) ❑	(7) ❑
Pasta/ris, vanlig type	(1) ❑	(8) ❑	(2) ❑	(3) ❑	(9) ❑	(4) ❑	(5) ❑	(6) ❑	(7) ❑
Pasta/ris, fullkornstype	(1) ❑	(8) ❑	(2) ❑	(3) ❑	(9) ❑	(4) ❑	(5) ❑	(6) ❑	(7) ❑

Hvor ofte spiser barnet følgende mat nå for tiden?

	Aldri/ikke forsøkt	Under 1 g/uke	1-2 g/uke	3-4 g/uke	5-6 g/uke	1 g/døgn	2 g/døgn	3 g/døgn	4 g eller mer/døgn
Kaker, vafler, søt kjeks o.l.	(1) ❑	(8) ❑	(2) ❑	(3) ❑	(9) ❑	(4) ❑	(5) ❑	(6) ❑	(7) ❑
Dessert/iskrem	(1) ❑	(8) ❑	(2) ❑	(3) ❑	(9) ❑	(4) ❑	(5) ❑	(6) ❑	(7) ❑
Sjokolade	(1) ❑	(8) ❑	(2) ❑	(3) ❑	(9) ❑	(4) ❑	(5) ❑	(6) ❑	(7) ❑
Smågodt, seigmenn o.l.	(1) ❑	(8) ❑	(2) ❑	(3) ❑	(9) ❑	(4) ❑	(5) ❑	(6) ❑	(7) ❑
Chips/potetgull o.l.	(1) ❑	(8) ❑	(2) ❑	(3) ❑	(9) ❑	(4) ❑	(5) ❑	(6) ❑	(7) ❑

Hvor ofte spiser barnet følgende grønnsaker nå for tiden?
Ta med både rå, kokte og mosede grønnsaker

	Aldri/ikke forsøkt	Under 1 g/uke	1-2 g/uke	3-4 g/uke	5-6 g/uke	1 g/døgn	2 g/døgn	3 g/døgn	4 g eller mer/døgn
Potet	(1) ❑	(8) ❑	(2) ❑	(3) ❑	(9) ❑	(4) ❑	(5) ❑	(6) ❑	(7) ❑
Gulrot	(1) ❑	(8) ❑	(2) ❑	(3) ❑	(9) ❑	(4) ❑	(5) ❑	(6) ❑	(7) ❑
Kålrot	(1) ❑	(8) ❑	(2) ❑	(3) ❑	(9) ❑	(4) ❑	(5) ❑	(6) ❑	(7) ❑
Søtpotet	(1) ❑	(8) ❑	(2) ❑	(3) ❑	(9) ❑	(4) ❑	(5) ❑	(6) ❑	(7) ❑
Blomkål	(1) ❑	(8) ❑	(2) ❑	(3) ❑	(9) ❑	(4) ❑	(5) ❑	(6) ❑	(7) ❑
Brokkoli	(1) ❑	(8) ❑	(2) ❑	(3) ❑	(9) ❑	(4) ❑	(5) ❑	(6) ❑	(7) ❑
Grønn salat	(1) ❑	(8) ❑	(2) ❑	(3) ❑	(9) ❑	(4) ❑	(5) ❑	(6) ❑	(7) ❑
Spinat	(1) ❑	(8) ❑	(2) ❑	(3) ❑	(9) ❑	(4) ❑	(5) ❑	(6) ❑	(7) ❑

Hvor ofte spiser barnet følgende grønnsaker nå for tiden?
Ta med både rå, kokte og mosede grønnsaker

	Aldri/ikke forsøkt	Under 1 g/uke	1-2 g/uke	3-4 g/uke	5-6 g/uke	1 g/døgn	2 g/døgn	3 g/døgn	4 g eller mer/døgn
Agurk	(1) ❑	(8) ❑	(2) ❑	(3) ❑	(9) ❑	(4) ❑	(5) ❑	(6) ❑	(7) ❑
Tomat	(1) ❑	(8) ❑	(2) ❑	(3) ❑	(9) ❑	(4) ❑	(5) ❑	(6) ❑	(7) ❑
Mais	(1) ❑	(8) ❑	(2) ❑	(3) ❑	(9) ❑	(4) ❑	(5) ❑	(6) ❑	(7) ❑

	Aldri/ikke forsøkt	Under 1 g/uke	1-2 g/uke	3-4 g/uke	5-6 g/uke	1 g/døgn	2 g/døgn	3 g/døgn	4 g eller mer/døgn
Paprika	(1) ❑	(8) ❑	(2) ❑	(3) ❑	(9) ❑	(4) ❑	(5) ❑	(6) ❑	(7) ❑
Erter/bønner	(1) ❑	(8) ❑	(2) ❑	(3) ❑	(9) ❑	(4) ❑	(5) ❑	(6) ❑	(7) ❑
Frossen grønnsaksblanding	(1) ❑	(8) ❑	(2) ❑	(3) ❑	(9) ❑	(4) ❑	(5) ❑	(6) ❑	(7) ❑
Råkost	(1) ❑	(8) ❑	(2) ❑	(3) ❑	(9) ❑	(4) ❑	(5) ❑	(6) ❑	(7) ❑

Hvor ofte spiser barnet følgende frukt og bær nå for tiden?

	Aldri/ikke forsøkt	Under 1 g/uke	1-2 g/uke	3-4 g/uke	5-6 g/uke	1 g/døgn	2 g/døgn	3 g/døgn	4 g eller mer/døgn
Appelsin/klementin o.l.	(1) ❑	(8) ❑	(2) ❑	(3) ❑	(9) ❑	(4) ❑	(5) ❑	(6) ❑	(7) ❑
Banan	(1) ❑	(8) ❑	(2) ❑	(3) ❑	(9) ❑	(4) ❑	(5) ❑	(6) ❑	(7) ❑
Eple	(1) ❑	(8) ❑	(2) ❑	(3) ❑	(9) ❑	(4) ❑	(5) ❑	(6) ❑	(7) ❑
Pære	(1) ❑	(8) ❑	(2) ❑	(3) ❑	(9) ❑	(4) ❑	(5) ❑	(6) ❑	(7) ❑
Plomme	(1) ❑	(8) ❑	(2) ❑	(3) ❑	(9) ❑	(4) ❑	(5) ❑	(6) ❑	(7) ❑
Druer	(1) ❑	(8) ❑	(2) ❑	(3) ❑	(9) ❑	(4) ❑	(5) ❑	(6) ❑	(7) ❑

Hvor ofte spiser barnet følgende frukt og bær nå for tiden?

	Aldri/ikke forsøkt	Under 1 g/uke	1-2 g/uke	3-4 g/uke	5-6 g/uke	1 g/døgn	2 g/døgn	3 g/døgn	4 g eller mer/døgn
Kiwi	(1) ❑	(8) ❑	(2) ❑	(3) ❑	(9) ❑	(4) ❑	(5) ❑	(6) ❑	(7) ❑
Melon	(1) ❑	(8) ❑	(2) ❑	(3) ❑	(9) ❑	(4) ❑	(5) ❑	(6) ❑	(7) ❑
Mango	(1) ❑	(8) ❑	(2) ❑	(3) ❑	(9) ❑	(4) ❑	(5) ❑	(6) ❑	(7) ❑
Bær, alle typer	(1) ❑	(8) ❑	(2) ❑	(3) ❑	(9) ❑	(4) ❑	(5) ❑	(6) ❑	(7) ❑

Får barnet hjemmelaget middagsmat eller ferdigkjøpt (industrifremstilt) barnemat på glass eller pose?

(1) ❑ Bare hjemmelaget

(2) ❑ Mest hjemmelaget

(3) ❑ Omtrent halvparten av hvert

(4) ❑ Mest ferdigkjøpt

(5) ❑ Bare ferdigkjøpt

Får barnet tran eller vitamintilskudd?

(1) ❑ Ja

(2) ❑ Nei

Angi hva slags type kosttilskudd, mengde og hyppighet.

	**Antall t-skjeer per gang?**					**Hvor ofte?**		
	**Ikke aktuelt**	**1**	**2**	**3**	**4 eller fler**	**Ikke aktuelt**	**daglig**	**av og til**
Tran	(1) ❑	(2) ❑	(5) ❑	(4) ❑	(3) ❑	(1) ❑	(2) ❑	(3) ❑
Flytende vitamin- / multivitamintilskudd	(1) ❑	(2) ❑	(5) ❑	(4) ❑	(3) ❑	(1) ❑	(2) ❑	(3) ❑

Hva slags konsistens er det på maten barnet ditt spiser nå?

	**Spiser ofte**	**Spiser noen ganger**	**Spiser sjelden**	**Spiser aldri/spiste før**	**Aldri tilbudt/forsøkt**
Pureer/helt glatt mat	(1) ❑	(2) ❑	(3) ❑	(5) ❑	(4) ❑
Finmost mat	(1) ❑	(2) ❑	(3) ❑	(5) ❑	(4) ❑
Most mat med klumper	(1) ❑	(2) ❑	(3) ❑	(5) ❑	(4) ❑
Finhakket/kuttet mat	(1) ❑	(2) ❑	(3) ❑	(5) ❑	(4) ❑
Fingermat/mat i biter	(1) ❑	(2) ❑	(3) ❑	(5) ❑	(4) ❑

Nå følger noen spørsmål om barnets matlyst og spisevaner. Tenk på hvordan det vanligvis pleier å være når du svarer på spørsmålene. Noen av spørsmålene kan likne hverandre, men de belyser litt ulike tema og det er fint om du svarer på alle spørsmålene.

Les utsagnene under og kryss av for det svaralternativet som passer best på ditt barns spiseatferd

	Aldri	Sjelden	Noen ganger	Ofte	Alltid
Barnet mitt elsker mat	(1) ❑	(2) ❑	(3) ❑	(4) ❑	(5) ❑
Barnet mitt spiser mer når hun/han er bekymret	(1) ❑	(2) ❑	(3) ❑	(4) ❑	(5) ❑
Barnet mitt har stor appetitt	(1) ❑	(2) ❑	(3) ❑	(4) ❑	(5) ❑
Barnet mitt bruker kort tid på måltidet	(1) ❑	(2) ❑	(3) ❑	(4) ❑	(5) ❑
Barnet mitt er interessert i mat	(1) ❑	(2) ❑	(3) ❑	(4) ❑	(5) ❑
Barnet mitt spør alltid om noe å drikke	(1) ❑	(2) ❑	(3) ❑	(4) ❑	(5) ❑
Barnet mitt nekter å spise ny mat de første gangene det tilbys	(1) ❑	(2) ❑	(3) ❑	(4) ❑	(5) ❑
Barnet mitt spiser sakte	(1) ❑	(2) ❑	(3) ❑	(4) ❑	(5) ❑
Barnet mitt spiser mindre når hun/han er sint	(1) ❑	(2) ❑	(3) ❑	(4) ❑	(5) ❑

Les utsagnene under og kryss av for det svaralternativet som passer best på ditt barns spiseatferd

	Aldri	Sjelden	Noen ganger	Ofte	Alltid
Barnet mitt liker å smake ny mat	(1) ❑	(2) ❑	(3) ❑	(4) ❑	(5) ❑
Barnet mitt spiser mindre når hun/han er trøtt	(1) ❑	(2) ❑	(3) ❑	(4) ❑	(5) ❑
Barnet mitt ber alltid om mat	(1) ❑	(2) ❑	(3) ❑	(4) ❑	(5) ❑
Barnet mitt spiser mer når hun/han er irritert	(1) ❑	(2) ❑	(3) ❑	(4) ❑	(5) ❑
Hvis hun/han fikk lov, ville barnet mitt spise for mye	(1) ❑	(2) ❑	(3) ❑	(4) ❑	(5) ❑
Barnet mitt spiser mer når hun/han er engstelig	(1) ❑	(2) ❑	(3) ❑	(4) ❑	(5) ❑

	Aldri	Sjelden	Noen ganger	Ofte	Alltid
Barnet mitt liker mye forskjellig mat	(1) ❑	(2) ❑	(3) ❑	(4) ❑	(5) ❑
Barnet mitt spiser ikke opp maten sin	(1) ❑	(2) ❑	(3) ❑	(4) ❑	(5) ❑
Barnet mitt bruker mer enn 30 min. på å bli ferdig med et måltid	(1) ❑	(2) ❑	(3) ❑	(4) ❑	(5) ❑

Les utsagnene under og kryss av for det svaralternativet som passer best på ditt barns spiseatferd

	Aldri	Sjelden	Noen ganger	Ofte	Alltid
Hvis barnet mitt fikk velge, så ville hun/han spise det meste av tiden	(1) ❑	(2) ❑	(3) ❑	(4) ❑	(5) ❑
Barnet mitt gleder seg til måltidene	(1) ❑	(2) ❑	(3) ❑	(4) ❑	(5) ❑
Barnet mitt blir mett før hun/han har spist opp	(1) ❑	(2) ❑	(3) ❑	(4) ❑	(5) ❑
Barnet mitt liker å spise	(1) ❑	(2) ❑	(3) ❑	(4) ❑	(5) ❑
Barnet mitt spiser mer når hun/han er glad	(1) ❑	(2) ❑	(3) ❑	(4) ❑	(5) ❑
Barnet mitt er sjelden fornøyd med maten hun/han får servert	(1) ❑	(2) ❑	(3) ❑	(4) ❑	(5) ❑
Barnet mitt spiser mindre når hun/han er lei seg eller opprørt	(1) ❑	(2) ❑	(3) ❑	(4) ❑	(5) ❑
Barnet mitt blir fort mett	(1) ❑	(2) ❑	(3) ❑	(4) ❑	(5) ❑
Barnet mitt spiser mer når hun/han ikke har noe å gjøre	(1) ❑	(2) ❑	(3) ❑	(4) ❑	(5) ❑

Les utsagnene under og kryss av for det svaralternativet som passer best på ditt barns spiseatferd

	Aldri	Sjelden	Noen ganger	Ofte	Alltid
Selv om barnet mitt er mett, så har hun/han plass til favorittmaten sin	(1) ❑	(2) ❑	(3) ❑	(4) ❑	(5) ❑
Hvis hun/han fikk muligheten, så ville barnet mitt drikke hele dagen lang	(1) ❑	(2) ❑	(3) ❑	(4) ❑	(5) ❑
Barnet mitt klarer ikke å spise et vanlig måltid hvis hun/han har spist et mellom-måltid like før	(1) ❑	(2) ❑	(3) ❑	(4) ❑	(5) ❑
Hvis hun/han fikk muligheten, så ville barnet mitt alltid hatt noe å drikke	(1) ❑	(2) ❑	(3) ❑	(4) ❑	(5) ❑
Barnet mitt er interessert i å smake mat som hun/han ikke har smakt før	(1) ❑	(2) ❑	(3) ❑	(4) ❑	(5) ❑
Barnet mitt bestemmer seg for at hun/han ikke liker enkelte matsorter, selv uten å ha smakt	(1) ❑	(2) ❑	(3) ❑	(4) ❑	(5) ❑
Hvis hun/han fikk muligheten, så ville barnet mitt hatt mat i munnen hele tiden	(1) ❑	(2) ❑	(3) ❑	(4) ❑	(5) ❑
Barnet mitt spiser stadig saktere utover i måltidet	(1) ❑	(2) ❑	(3) ❑	(4) ❑	(5) ❑

Hvor enig er du i følgende påstander?

	Svært uenig	Noe uenig	Litt enig	Veldig enig
Barnet mitt stoler ikke på ukjent mat	(1) ❑	(2) ❑	(3) ❑	(4) ❑
Hvis barnet mitt ikke vet hva som er i maten, vil hun/han ikke smake	(1) ❑	(2) ❑	(3) ❑	(4) ❑

	Svært uenig	Noe uenig	Litt enig	Veldig enig
Barnet mitt er redd for å spise noe hun/han ikke har spist før	(1) ❑	(2) ❑	(3) ❑	(4) ❑
Barnet mitt vil spise nesten all slags mat	(1) ❑	(2) ❑	(3) ❑	(4) ❑
Barnet mitt er veldig kresen på hva slags mat hun/han vil spise	(1) ❑	(2) ❑	(3) ❑	(4) ❑
Barnet mitt prøver stadig ny og ukjent mat	(1) ❑	(2) ❑	(3) ❑	(4) ❑

Hvor mange ganger tilbyr du en ny matvare til barnet før du vurderer at barnet ikke liker maten?

(1) ❑ En gang
(6) ❑ To ganger
(5) ❑ 3-5 ganger
(2) ❑ 6-10 ganger
(3) ❑ 11 ganger eller mer

Nå kommer noen spørsmål om barnets matlyst og hvordan det oppleves å gi mat til barnet. Tenk på hvordan det har pleid å være den siste måneden når du svarer på spørsmålene.

Hvor enig eller uenig er du i følgende utsagn:

	Helt uenig	Litt uenig	Både og	Litt enig	Helt enig
Jeg må forsikre meg om at barnet ikke spiser for mye søtsaker (f.eks. godteri, is eller kake, søt kjeks)	(1) ❑	(2) ❑	(3) ❑	(4) ❑	(5) ❑
Jeg må forsikre meg om at barnet ikke spiser for mye fet mat	(1) ❑	(2) ❑	(3) ❑	(4) ❑	(5) ❑
Jeg må forsikre meg om at	(1) ❑	(2) ❑	(3) ❑	(4) ❑	(5) ❑

	Helt uenig	Litt uenig	Både og	Litt enig	Helt enig
barnet ikke spiser for mye av hans/ henne favorittmat					
Jeg holder med hensikt noen typer mat unna barnet	(1) ❑	(2) ❑	(3) ❑	(4) ❑	(5) ❑
Jeg gir søtsaker (f.eks. godteri, iskrem eller kaker) til barnet som belønning for god oppførsel	(1) ❑	(2) ❑	(3) ❑	(4) ❑	(5) ❑
Jeg tilbyr barnet hans/ hennes favorittmat som belønning for god oppførsel	(1) ❑	(2) ❑	(3) ❑	(4) ❑	(5) ❑

Hvor enig eller uenig er du i følgende utsagn:

	Helt uenig	Litt uenig	Både og	Litt enig	Helt enig
Hvis jeg ikke regulerer barnets spising, ville han/ hun spist alt for mye usunn mat	(1) ❑	(2) ❑	(3) ❑	(4) ❑	(5) ❑
Hvis jeg ikke regulerer barnets spising, ville han/ hun spist alt for mye av sin favorittmat	(1) ❑	(2) ❑	(3) ❑	(4) ❑	(5) ❑
Barnet bør (skal) alltid spise opp all maten på tallerken sin	(1) ❑	(2) ❑	(3) ❑	(4) ❑	(5) ❑
Jeg må passe spesielt på for å være sikker på at barnet spiser nok	(1) ❑	(2) ❑	(3) ❑	(4) ❑	(5) ❑
Hvis barnet sier «jeg er ikke sulten» prøver jeg likevel å få han/ henne til å spise	(1) ❑	(2) ❑	(3) ❑	(4) ❑	(5) ❑
Hvis jeg ikke regulerte barnets spising ville han/ hun spist alt for lite	(1) ❑	(2) ❑	(3) ❑	(4) ❑	(5) ❑

Og hvor ofte følger du med på..

	Aldri	Sjelden	Noen ganger	Ofte	Alltid
..hvor mye søtsaker (godterier, is, kaker/kjeks o.l.) som barnet ditt spiser	(1) ❑	(2) ❑	(3) ❑	(4) ❑	(5) ❑
..hvor mye snacks (potetchips, ostepop o.l.) som barnet ditt spiser?	(1) ❑	(2) ❑	(3) ❑	(4) ❑	(5) ❑
..hvor mye fet mat som barnet ditt spiser?	(1) ❑	(2) ❑	(3) ❑	(4) ❑	(5) ❑

Her følger noen flere spørsmål om barnets spisevaner og matlyst og hvordan du forholder deg til det. Det er ingen "riktige" eller "gale" svar, vi er interessert i hva du som forelder virkelig føler og gjør.

Hvor ofte stemmer utsagnene under?

	Aldri	Sjelden	Noen ganger	Ofte	Alltid
Jeg lar barnet mitt velge hva slags mat hun/han vil ha til måltider	(1) ❑	(2) ❑	(3) ❑	(4) ❑	(5) ❑
Jeg gir barnet mitt noe å spise for å føle seg bedre når hun/han føler seg opprørt	(1) ❑	(2) ❑	(3) ❑	(4) ❑	(5) ❑
Jeg oppmuntrer barnet mitt til å glede seg til måltidet	(1) ❑	(2) ❑	(3) ❑	(4) ❑	(5) ❑
Jeg roser barnet mitt hvis hun/han spiser det jeg gir henne/han	(1) ❑	(2) ❑	(3) ❑	(4) ❑	(5) ❑
Jeg bestemmer hvor mye snacks barnet mitt skal få	(1) ❑	(2) ❑	(3) ❑	(4) ❑	(5) ❑
Jeg oppmuntrer barnet mitt til å spise mye forskjellig og	(1) ❑	(2) ❑	(3) ❑	(4) ❑	(5) ❑

	Aldri	Sjelden	Noen ganger	Ofte	Alltid
variert mat					
For å få barnet mitt til å oppføre seg fint, lover jeg henne/han noe å spise	(1) ❑	(2) ❑	(3) ❑	(4) ❑	(5) ❑
Jeg presenterer mat på en attraktiv måte for barnet mitt	(1) ❑	(2) ❑	(3) ❑	(4) ❑	(5) ❑
Hvis barnet mitt oppfører seg dårlig, holder jeg tilbake favorittmaten hennes/hans	(1) ❑	(2) ❑	(3) ❑	(4) ❑	(5) ❑

Hvor ofte stemmer utsagnene under?

	Aldri	Sjelden	Noen ganger	Ofte	Alltid
Jeg oppmuntrer barnet mitt til å smake på alle mattypene jeg serverer til måltider	(1) ❑	(2) ❑	(3) ❑	(4) ❑	(5) ❑
Jeg tillater barnet mitt å vandre rundt under måltidet	(1) ❑	(2) ❑	(3) ❑	(4) ❑	(5) ❑
Jeg oppmuntrer barnet mitt til å smake på mat hun/han ikke har smakt før	(1) ❑	(2) ❑	(3) ❑	(4) ❑	(5) ❑
Jeg gir barnet mitt noe å spise for å føle seg bedre når hun/han har slått seg	(1) ❑	(2) ❑	(3) ❑	(4) ❑	(5) ❑
Jeg lar barnet mitt bestemme når hun/han vil spise måltidet sitt	(1) ❑	(2) ❑	(3) ❑	(4) ❑	(5) ❑
Jeg gir barnet mitt noe å spise hvis hun/han kjeder seg	(1) ❑	(2) ❑	(3) ❑	(4) ❑	(5) ❑

	Aldri	Sjelden	Noen ganger	Ofte	Alltid
Jeg lar barnet mitt bestemme når hun/han har spist nok snacks	(1) ❑	(2) ❑	(3) ❑	(4) ❑	(5) ❑
Jeg bestemmer når det er tid for at barnet mitt kan spise en matbit/snack	(1) ❑	(2) ❑	(3) ❑	(4) ❑	(5) ❑
Jeg bruker dessert som belønning for å få barnet mitt til å spise middag	(1) ❑	(2) ❑	(3) ❑	(4) ❑	(5) ❑

Hvor ofte stemmer utsagnene under?

	Aldri	Sjelden	Noen ganger	Ofte	Alltid
Jeg oppmuntrer barnet mitt til å sette pris på maten sin	(1) ❑	(2) ❑	(3) ❑	(4) ❑	(5) ❑
Jeg bestemmer tidspunktene for når barnet mitt spiser måltidene sine	(1) ❑	(2) ❑	(3) ❑	(4) ❑	(5) ❑
Jeg gir barnet mitt noe å spise for å føle seg bedre når hun/han er bekymret	(1) ❑	(2) ❑	(3) ❑	(4) ❑	(5) ❑
Jeg belønner barnet mitt med noe å spise når hun/han oppfører seg fint	(1) ❑	(2) ❑	(3) ❑	(4) ❑	(5) ❑
Jeg lar barnet mitt spise mellom måltidene når hun/han vil	(1) ❑	(2) ❑	(3) ❑	(4) ❑	(5) ❑
Jeg insisterer på at barnet mitt spiser måltider ved	(1) ❑	(2) ❑	(3) ❑	(4) ❑	(5) ❑

	Aldri	Sjelden	Noen ganger	Ofte	Alltid
bordet					
Jeg gir barnet mitt noe å spise for å føle seg bedre når hun/han er sint	(1) ❑	(2) ❑	(3) ❑	(4) ❑	(5) ❑
Jeg bestemmer hva barnet mitt spiser mellom måltider	(1) ❑	(2) ❑	(3) ❑	(4) ❑	(5) ❑
Jeg roser barnet mitt når hun/han spiser ny mat	(1) ❑	(2) ❑	(3) ❑	(4) ❑	(5) ❑

Nå kommer andre del av spørreskjemaet med spørsmål om deg som er mor eller far. Spørsmålene omhandler bakgrunnsopplysninger, matvaner og vurdering av egen fysisk og psykisk helse.

Hva er din fødselsdato?
Må skrives år-mnd-dato, f.eks, 1988-07-03

______

Har du, ev. mor til barnet som deltar i undersøkelsen, blitt gravid på ny etter at barnet som deltar i undersøkelsen ble født?

(1) ❑ Nei
(2) ❑ Ja, jeg er gravid nå
(3) ❑ Ja, jeg har født barnet

Bor du sammen med barnets andre forelder?
(1) ❑ Ja
(2) ❑ Nei

Hvilken sivilstand har du nå?

(1) ❑ Gift
(2) ❑ Samboer
(3) ❑ Enslig
(4) ❑ Skilt/separert
(5) ❑ Enke/enkemann
(6) ❑ Annet, beskriv _____

Hvilken utdannelse har du nå?
Velg høyeste fullførte utdanning

(1) ❑ Mindre enn 9/10 års grunnskole
(2) ❑ Grunnskole
(3) ❑ Videregående skole
(4) ❑ Videregående yrkesfag
(5) ❑ Universitet/høyskole inntil 4 år
(6) ❑ Universitet/høyskole mer enn 4 år
(7) ❑ Annen utdanning

Hva er din hovedaktivitet nå?

(1) ❑ Arbeid heltid
(2) ❑ Arbeid deltid
(3) ❑ Hjemmeværende
(4) ❑ Sykemeldt
(5) ❑ Permisjon
(6) ❑ Uføretrygdet
(7) ❑ Under attføring/rehabilitering
(8) ❑ Student/skoleelev
(9) ❑ Arbeidsledig
(10) ❑ Annet

Er økonomien slik at du/dere vil ha mulighet til å klare en uforutsett regning på kr. 3000 til f. eks. tannlege eller en reparasjon?

(1) ❑ Nei
(3) ❑ Ja

(2) ❑ Vet ikke

Har det i løpet av det siste året hendt at du/dere har hatt vansker med å klare løpende utgifter til mat, transport, husleie og lignende?

(1) ❑ Nei

(2) ❑ Ja

(3) ❑ Vet ikke

Nå følger noen spørsmål om levevaner og livsstil:

Hvor mye veier du nå?
Svar i antall kg

______

Hvor høy er du? Svar i antall cm

______

Røyker du?

(1) ❑ Nei, har aldri røykt regelmessig

(2) ❑ Nei, har sluttet

(3) ❑ Ja, men ikke daglig

(4) ❑ Ja, daglig

Snuser du?

(1) ❑ Nei, har aldri snust regelmessig

(2) ❑ Nei, har sluttet

(3) ❑ Ja, men ikke daglig

(4) ❑ Ja, daglig

Har du hovedansvaret for matlagingen hjemme?

(1) ❑ Ja

(2) ❑ Nei

(3) ❑ Ansvaret er delt

Hvor ofte gjør du følgende?

	Aldri	Mindre enn 1 g/u	1 g/u	2 g/u	3 g/u	4 g/u	5 g/u	6 g/u	Hver dag
Kutter opp grønnsaker	(1) ❑	(2) ❑	(3) ❑	(4) ❑	(5) ❑	(6) ❑	(7) ❑	(8) ❑	(9) ❑
Kutter opp frukt	(1) ❑	(2) ❑	(3) ❑	(4) ❑	(5) ❑	(6) ❑	(7) ❑	(8) ❑	(9) ❑
Lager middag fra bunnen	(1) ❑	(2) ❑	(3) ❑	(4) ❑	(5) ❑	(6) ❑	(7) ❑	(8) ❑	(9) ❑
Baker brød/rundstykker	(1) ❑	(2) ❑	(3) ❑	(4) ❑	(5) ❑	(6) ❑	(7) ❑	(8) ❑	(9) ❑

Da er du ferdig med å fylle ut skjemaet.
Du sender det inn ved å trykke på knappen under.

Tusen takk!
