# Supplementary material for: Examining the effects of an eHealth intervention from infant age 6 to 12 months on child eating behaviors and maternal feeding practices one year after cessation: The Norwegian randomized controlled trial Early Food for Future Health
Source: PLoS One. 2019 Aug 23;14(8):e0220437. doi: 10.1371/journal.pone.0220437 (PMC6707582; doi:10.1371/journal.pone.0220437)
Supplement: S2 File — (PDF) [file pone.0220437.s004.pdf]

Thank you very much for still taking part in the Baby e-Food Study!

It's easiest to fill out the questionnaire on a computer. You navigate in the form by pressing the arrows / "next button" at the bottom of the page.

After some initial questions, the questionnaire is two-fold:
The first part of the questionnaire is about the child participating in the survey. The second part is about you who is the mother or father of the child.

Good luck!

What is your relationship with the child participating in the survey?

(1) ❑ Mother
(2) ❑ Father
(3) ❑ None, describe _____

What is the child´s date of birth?
Must be written DD.MM:YYYY

____

What is the child's gender?

(1) ❑ girl
(2) ❑ boy

Is the child participating in this study your only child?

(0) ❑ Yes
(1) ❑ No

How many children do you have?

(1) ❑ Two
(2) ❑ Three
(3) ❑ Four or more

Were you in the group who had access to the website and movies? (Intervention group)

(1) ❑ Yes
(2) ❑ No

Did you watch the monthly infant-feeding videos?

(1) ❑ Yes, watched all the videos
(2) ❑ Yes, watched most of the videos
(3) ❑ Yes, watched about half of the videos
(4) ❑ Yes, watched one or two of the videos
(5) ❑ No, watched none of the videos

Did you watch the cooking films or recipes?

(1) ❑ Yes, watched all the cooking films/recipes
(2) ❑ Yes, watched most of the cooking films/recipes
(3) ❑ Yes, watched about half of the cooking films/recipes
(4) ❑ Yes, watched one or two of the cooking films/recipes
(5) ❑ No, watched none of the cooking films/recipes

Now follow some questions about the child´s growth and development over the past year

Based on the child's health card from the child health center, enter the date of examination, weight and length in the questions below. If you do not have the current weight or height measures, you can proceed with the form.

Examination at ap. 18 months of age:

Date of investigation _____

weight in grams _____

length in cm _____

Examination at ap. 24 months of age:

Date of investigation _____

weight in grams _____

length in cm _____

Now follow questions about the child's diet and eating at two years of age:

How often does your child eat the following meals within a week?

	Never / seldom	1 / week	2 / week	3 / week	4 / week	5 / week	6 / week	Every day
Breakfast	(1) ❑	(2) ❑	(3) ❑	(4) ❑	(5) ❑	(6) ❑	(7) ❑	(8) ❑
Lunch	(1) ❑	(2) ❑	(3) ❑	(4) ❑	(5) ❑	(6) ❑	(7) ❑	(8) ❑
Snack before dinner	(1) ❑	(2) ❑	(3) ❑	(4) ❑	(5) ❑	(6) ❑	(7) ❑	(8) ❑
Dinner	(1) ❑	(2) ❑	(3) ❑	(4) ❑	(5) ❑	(6) ❑	(7) ❑	(8) ❑
Snack after dinner	(1) ❑	(2) ❑	(3) ❑	(4) ❑	(5) ❑	(6) ❑	(7) ❑	(8) ❑
Supper	(1) ❑	(2) ❑	(3) ❑	(4) ❑	(5) ❑	(6) ❑	(7) ❑	(8) ❑
Other meals/snacks	(1) ❑	(2) ❑	(3) ❑	(4) ❑	(5) ❑	(6) ❑	(7) ❑	(8) ❑

How often does the child eat the following meals together with family now? (at least one adult eating the same meal)

	Aldri/sjelden	1-3 ganger per uke	4-6 ganger per uke	Hver dag
Breakfast	(1) ❑	(2) ❑	(3) ❑	(4) ❑
Lunch	(1) ❑	(2) ❑	(3) ❑	(4) ❑
Dinner	(1) ❑	(2) ❑	(3) ❑	(4) ❑
Supper	(1) ❑	(2) ❑	(3) ❑	(4) ❑

Is it you who usually gives the baby food?

(1) ❑ Yes

(2) ❑ No

(3) ❑ Share alike

Do you feed the child (ie, an adult keeps the spoon or splits the food and gives it bit by bit), or does he/she eats by him/herself

	Always eats by her / him self	Mostly eats by him/her self	Both	Mostly fed	Always fed
Breakfast	(1) ❑	(3) ❑	(4) ❑	(5) ❑	(2) ❑
Lunch	(1) ❑	(3) ❑	(4) ❑	(5) ❑	(2) ❑
Afternoon (meal after lunch and before dinner)	(1) ❑	(3) ❑	(4) ❑	(5) ❑	(2) ❑

	Always eats by her / him self	Mostly eats by him/her self	Both	Mostly fed	Always fed
Dinner	(1) ❑	(3) ❑	(4) ❑	(5) ❑	(2) ❑
Supper	(1) ❑	(3) ❑	(4) ❑	(5) ❑	(2) ❑
Other meals / snacks	(1) ❑	(3) ❑	(4) ❑	(5) ❑	(2) ❑

How often do the statements below match the child's meals now?

	Almost always	Often	Sometimes	Seldom	Almost never
The child eats the same for dinner as the rest of the family	(1) ❑	(2) ❑	(3) ❑	(4) ❑	(5) ❑
I / we make separate dinner for the child	(1) ❑	(2) ❑	(3) ❑	(4) ❑	(5) ❑
The child sits at the table when she / he eats	(1) ❑	(2) ❑	(3) ❑	(4) ❑	(5) ❑
The child plays or watches TV / tablet / smartphone while eating	(1) ❑	(2) ❑	(3) ❑	(4) ❑	(5) ❑

Has or has the child had problems with eating / eating?
Here you can make several crosses

(1) ❑ No, have no problems
(2) ❑ Yes, poor appetite / eats too little
(3) ❑ Yes, like few food types
(4) ❑ Yes, difficulties adjusting to the family diet
(5) ❑ Yes, allergy / intolerance to some food types
(6) ❑ Yes, other problems - describe _____

How often does the child have the following drink nowadays?

	Never / seldom	1-3 times per week	4-6 times per week	1 time a day	2 times a day	3 times a day	4 times a day	5 or more times a day
Milk, all types	(1) ❑	(2) ❑	(3) ❑	(4) ❑	(5) ❑	(6) ❑	(7) ❑	(8) ❑
Sour milk (e.g. yoghurt)	(1) ❑	(2) ❑	(3) ❑	(4) ❑	(5) ❑	(6) ❑	(7) ❑	(8) ❑
Chocolate milk, all types	(1) ❑	(2) ❑	(3) ❑	(4) ❑	(5) ❑	(6) ❑	(7) ❑	(8) ❑
Water	(1) ❑	(2) ❑	(3) ❑	(4) ❑	(5) ❑	(6) ❑	(7) ❑	(8) ❑

How often does the child have the following drink nowadays?

	Never / seldom	1-3 times per week	4-6 times per week	1 time a day	2 times a day	3 times a day	4 times a day	5 or more times a day
Limonade, with sugar	(1) ❑	(2) ❑	(3) ❑	(4) ❑	(5) ❑	(6) ❑	(7) ❑	(8) ❑
Limonade, sweetener	(1) ❑	(2) ❑	(3) ❑	(4) ❑	(5) ❑	(6) ❑	(7) ❑	(8) ❑
Juice	(1) ❑	(2) ❑	(3) ❑	(4) ❑	(5) ❑	(6) ❑	(7) ❑	(8) ❑
Mineral water with sugar	(1) ❑	(2) ❑	(3) ❑	(4) ❑	(5) ❑	(6) ❑	(7) ❑	(8) ❑
Mineral water, sweetener	(1) ❑	(2) ❑	(3) ❑	(4) ❑	(5) ❑	(6) ❑	(7) ❑	(8) ❑

How often does the child eat the following food nowadays?

How often?

	Never / seldom	1-3 /week	4-6 / week	1 /day	2 / day	3 / day	≥ 4 /day
Industrial-made porridge, all types	(1) ❑	(2) ❑	(3) ❑	(4) ❑	(5) ❑	(6) ❑	(7) ❑
Homemade porridge of whole-grain flour or oatmeal	(1) ❑	(2) ❑	(3) ❑	(4) ❑	(5) ❑	(6) ❑	(7) ❑
Homemade porridge of white flour, rusk, semule, rice, or corn	(1) ❑	(2) ❑	(3) ❑	(4) ❑	(5) ❑	(6) ❑	(7) ❑

How often does the child eat the following food nowadays?

How often?

	Never / not tried	< 1 time per week	1-2 times per week	3-4 times per week	5-6 times per week	1 time a day	2 times a day	3 times a day	≥ 4 times a day
Bread with liver paste	(1) ❑	(8) ❑	(2) ❑	(3) ❑	(9) ❑	(4) ❑	(5) ❑	(6) ❑	(7) ❑
Bread with other cold meats	(1) ❑	(8) ❑	(2) ❑	(3) ❑	(9) ❑	(4) ❑	(5) ❑	(6) ❑	(7) ❑
Bread with toppings of fish	(1) ❑	(8) ❑	(2) ❑	(3) ❑	(9) ❑	(4) ❑	(5) ❑	(6) ❑	(7) ❑
Bread with cheese	(1) ❑	(8) ❑	(2) ❑	(3) ❑	(9) ❑	(4) ❑	(5) ❑	(6) ❑	(7) ❑

	How often?								
	**Never / not tried**	**< 1 time per week**	**1-2 times per week**	**3-4 times per week**	**5-6 times per week**	**1 time a day**	**2 times a day**	**3 times a day**	**≥ 4 times a day**
Bread with jam / honey	(1) ❑	(8) ❑	(2) ❑	(3) ❑	(9) ❑	(4) ❑	(5) ❑	(6) ❑	(7) ❑
Bread with other toppings	(1) ❑	(8) ❑	(2) ❑	(3) ❑	(9) ❑	(4) ❑	(5) ❑	(6) ❑	(7) ❑

How often does the child eat the following food nowadays?

	**Never / not tried**	**< 1 time per week**	**1-2 times per week**	**3-4 times per week**	**5-6 times per week**	**1 time a day**	**2 times a day**	**3 times a day**	**≥ 4 times a day**
Meat, minced meat, meatballs, sausage etc.	(1) ❑	(8) ❑	(2) ❑	(3) ❑	(9) ❑	(4) ❑	(5) ❑	(6) ❑	(7) ❑
Fish,fish caces, fish fingers etc.	(1) ❑	(8) ❑	(2) ❑	(3) ❑	(9) ❑	(4) ❑	(5) ❑	(6) ❑	(7) ❑
Pancakes	(1) ❑	(8) ❑	(2) ❑	(3) ❑	(9) ❑	(4) ❑	(5) ❑	(6) ❑	(7) ❑
Pizza	(1) ❑	(8) ❑	(2) ❑	(3) ❑	(9) ❑	(4) ❑	(5) ❑	(6) ❑	(7) ❑
Pasta / rice, ordinary type	(1) ❑	(8) ❑	(2) ❑	(3) ❑	(9) ❑	(4) ❑	(5) ❑	(6) ❑	(7) ❑
Pasta / rice, wholegrain	(1) ❑	(8) ❑	(2) ❑	(3) ❑	(9) ❑	(4) ❑	(5) ❑	(6) ❑	(7) ❑

How often does the child eat the following food nowadays?

	**Never / not tried**	**< 1 time per week**	**1-2 times per week**	**3-4 times per week**	**5-6 times per week**	**1 time a day**	**2 times a day**	**3 times a day**	**≥ 4 times a day**
Cakes, waffles, sweet biscuits	(1) ❑	(8) ❑	(2) ❑	(3) ❑	(9) ❑	(4) ❑	(5) ❑	(6) ❑	(7) ❑
Dessert / Ice Cream	(1) ❑	(8) ❑	(2) ❑	(3) ❑	(9) ❑	(4) ❑	(5) ❑	(6) ❑	(7) ❑
Chocolate	(1) ❑	(8) ❑	(2) ❑	(3) ❑	(9) ❑	(4) ❑	(5) ❑	(6) ❑	(7) ❑
Sweets / candy	(1) ❑	(8) ❑	(2) ❑	(3) ❑	(9) ❑	(4) ❑	(5) ❑	(6) ❑	(7) ❑
Chips	(1) ❑	(8) ❑	(2) ❑	(3) ❑	(9) ❑	(4) ❑	(5) ❑	(6) ❑	(7) ❑

How often does the child eat the following vegetables nowadays? Include both raw, cooked and mashed vegetables

	Never / not tried	< 1 time per week	1-2 times per week	3-4 times per week	5-6 times per week	1 time a day	2 times a day	3 times a day	≥ 4 times a day
Potato	(1) ❑	(8) ❑	(2) ❑	(3) ❑	(9) ❑	(4) ❑	(5) ❑	(6) ❑	(7) ❑
Carrot	(1) ❑	(8) ❑	(2) ❑	(3) ❑	(9) ❑	(4) ❑	(5) ❑	(6) ❑	(7) ❑
Rutabaga	(1) ❑	(8) ❑	(2) ❑	(3) ❑	(9) ❑	(4) ❑	(5) ❑	(6) ❑	(7) ❑
Sweet potato	(1) ❑	(8) ❑	(2) ❑	(3) ❑	(9) ❑	(4) ❑	(5) ❑	(6) ❑	(7) ❑
Cauliflower	(1) ❑	(8) ❑	(2) ❑	(3) ❑	(9) ❑	(4) ❑	(5) ❑	(6) ❑	(7) ❑
Broccoli	(1) ❑	(8) ❑	(2) ❑	(3) ❑	(9) ❑	(4) ❑	(5) ❑	(6) ❑	(7) ❑
Green salad	(1) ❑	(8) ❑	(2) ❑	(3) ❑	(9) ❑	(4) ❑	(5) ❑	(6) ❑	(7) ❑
Spinach	(1) ❑	(8) ❑	(2) ❑	(3) ❑	(9) ❑	(4) ❑	(5) ❑	(6) ❑	(7) ❑

How often does the child eat the following vegetables nowadays? Include both raw, cooked and mashed vegetables

	Never / not tried	< 1 time per week	1-2 times per week	3-4 times per week	5-6 times per week	1 time a day	2 times a day	3 times a day	≥ 4 times a day
Cucumber	(1) ❑	(8) ❑	(2) ❑	(3) ❑	(9) ❑	(4) ❑	(5) ❑	(6) ❑	(7) ❑
Tomato	(1) ❑	(8) ❑	(2) ❑	(3) ❑	(9) ❑	(4) ❑	(5) ❑	(6) ❑	(7) ❑
Corn	(1) ❑	(8) ❑	(2) ❑	(3) ❑	(9) ❑	(4) ❑	(5) ❑	(6) ❑	(7) ❑
Paprika	(1) ❑	(8) ❑	(2) ❑	(3) ❑	(9) ❑	(4) ❑	(5) ❑	(6) ❑	(7) ❑
Peas / beans	(1) ❑	(8) ❑	(2) ❑	(3) ❑	(9) ❑	(4) ❑	(5) ❑	(6) ❑	(7) ❑
Frozen vegetable mix	(1) ❑	(8) ❑	(2) ❑	(3) ❑	(9) ❑	(4) ❑	(5) ❑	(6) ❑	(7) ❑
Salad of raw vegetables	(1) ❑	(8) ❑	(2) ❑	(3) ❑	(9) ❑	(4) ❑	(5) ❑	(6) ❑	(7) ❑

How often does the child eat the following fruits and berries nowadays?

	Never / not tried	< 1 time per week	1-2 times per week	3-4 times per week	5-6 times per week	1 time a day	2 times a day	3 times a day	≥ 4 times a day

	Never / not tried	< 1 time per week	1-2 times per week	3-4 times per week	5-6 times per week	1 time a day	2 times a day	3 times a day	≥ 4 times a day
Orange / clementine	(1) ❑	(8) ❑	(2) ❑	(3) ❑	(9) ❑	(4) ❑	(5) ❑	(6) ❑	(7) ❑
Banana	(1) ❑	(8) ❑	(2) ❑	(3) ❑	(9) ❑	(4) ❑	(5) ❑	(6) ❑	(7) ❑
Apple	(1) ❑	(8) ❑	(2) ❑	(3) ❑	(9) ❑	(4) ❑	(5) ❑	(6) ❑	(7) ❑
Pear	(1) ❑	(8) ❑	(2) ❑	(3) ❑	(9) ❑	(4) ❑	(5) ❑	(6) ❑	(7) ❑
Plum	(1) ❑	(8) ❑	(2) ❑	(3) ❑	(9) ❑	(4) ❑	(5) ❑	(6) ❑	(7) ❑
Grapes	(1) ❑	(8) ❑	(2) ❑	(3) ❑	(9) ❑	(4) ❑	(5) ❑	(6) ❑	(7) ❑

How often does the child eat the following fruits and berries nowadays?

	Never / not tried	< 1 time per week	1-2 times per week	3-4 times per week	5-6 times per week	1 time a day	2 times a day	3 times a day	≥ 4 times a day
Kiwi	(1) ❑	(8) ❑	(2) ❑	(3) ❑	(9) ❑	(4) ❑	(5) ❑	(6) ❑	(7) ❑
Melon	(1) ❑	(8) ❑	(2) ❑	(3) ❑	(9) ❑	(4) ❑	(5) ❑	(6) ❑	(7) ❑
Mangos	(1) ❑	(8) ❑	(2) ❑	(3) ❑	(9) ❑	(4) ❑	(5) ❑	(6) ❑	(7) ❑
Berries, all types	(1) ❑	(8) ❑	(2) ❑	(3) ❑	(9) ❑	(4) ❑	(5) ❑	(6) ❑	(7) ❑

Does the child get homemade dinner or industrially made baby food on glass or bag?

(1) ☐ Only homemade
(2) ☐ Mostly homemade
(3) ☐ About half of each
(4) ☐ Mostly industrially made
(5) ☐ Only industrially made

Does the child get cod liver oil or other supplements?

(1) ❑ yes
(2) ❑ no

Specify type of dietary supplement, amount and frequency.

	Number of T-spoons?	How often?

	Not applicable	1	2	3	≥4	Not applicable	daily	Now and then
Cod liver oil	(1) ❑	(2) ❑	(5) ❑	(4) ❑	(3) ❑	(1) ❑	(2) ❑	(3) ❑
Fluent multivitamin supplement	(1) ❑	(2) ❑	(5) ❑	(4) ❑	(3) ❑	(1) ❑	(2) ❑	(3) ❑
Other	(1) ❑	(2) ❑	(5) ❑	(4) ❑	(3) ❑	(1) ❑	(2) ❑	(3) ❑

What is the consistency of the food your child is eating now?

	Eats often	Eats sometimes	Will not eat	Used to eat	Never tried / offered
Purees	(1) ❑	(2) ❑	(3) ❑	(4) ❑	(5) ❑
Fine-mashed food	(1) ❑	(2) ❑	(3) ❑	(4) ❑	(5) ❑
Mashed food with lumps	(1) ❑	(2) ❑	(3) ❑	(4) ❑	(5) ❑
Finely chopped / cut food	(1) ❑	(2) ❑	(3) ❑	(4) ❑	(5) ❑
Finger food / food in pieces	(1) ❑	(2) ❑	(3) ❑	(4) ❑	(5) ❑

Here are some questions about the child's appetite and eating habits. Think about how it usually tends to be when answering the questions. Some of the questions may resemble each other, but they highlight some different issues and it is great if you will answer all the questions.

Please read the following statements and tick the boxes most appropriate to your child's eating behaviour

	Never	Rarely	Some-times	Often	Always
My child loves food	(1) ❑	(2) ❑	(3) ❑	(4) ❑	(5) ❑
My child eats more when worried	(1) ❑	(2) ❑	(3) ❑	(4) ❑	(5) ❑
My child has a big appetite	(1) ❑	(2) ❑	(3) ❑	(4) ❑	(5) ❑
My child finishes his/her meal quickly	(1) ❑	(2) ❑	(3) ❑	(4) ❑	(5) ❑
My child is interested in food	(1) ❑	(2) ❑	(3) ❑	(4) ❑	(5) ❑
My child is always asking for a drink	(1) ❑	(2) ❑	(3) ❑	(4) ❑	(5) ❑

	Never	Rarely	Some-times	Often	Always
My child refuses new foods at first	(1) ❑	(2) ❑	(3) ❑	(4) ❑	(5) ❑
My child eats slowly	(1) ❑	(2) ❑	(3) ❑	(4) ❑	(5) ❑
My child eats less when angry	(1) ❑	(2) ❑	(3) ❑	(4) ❑	(5) ❑

Please read the following statements and tick the boxes most appropriate to your child's eating behaviour

	Never	Rarely	Some-times	Often	Always
My child enjoys tasting new foods	(1) ❑	(2) ❑	(3) ❑	(4) ❑	(5) ❑
My child eats less when s/he is tired	(1) ❑	(2) ❑	(3) ❑	(4) ❑	(5) ❑
My child is always asking for food	(1) ❑	(2) ❑	(3) ❑	(4) ❑	(5) ❑
My child eats more when annoyed	(1) ❑	(2) ❑	(3) ❑	(4) ❑	(5) ❑
If allowed to, my child would eat too much	(1) ❑	(2) ❑	(3) ❑	(4) ❑	(5) ❑
My child eats more when anxious	(1) ❑	(2) ❑	(3) ❑	(4) ❑	(5) ❑
My child enjoys a wide variety of foods	(1) ❑	(2) ❑	(3) ❑	(4) ❑	(5) ❑
My child leaves food on his/her plate at the end of a meal	(1) ❑	(2) ❑	(3) ❑	(4) ❑	(5) ❑
My child takes more than 30 minutes to finish a meal	(1) ❑	(2) ❑	(3) ❑	(4) ❑	(5) ❑

Please read the following statements and tick the boxes most appropriate to your child's eating behaviour

	Never	Rarely	Some-times	Often	Always
Given the choice, my child would eat most of the time	(1) ❑	(2) ❑	(3) ❑	(4) ❑	(5) ❑
My child looks forward to	(1) ❑	(2) ❑	(3) ❑	(4) ❑	(5) ❑

	Never	Rarely	Some-times	Often	Always
mealtimes					
My child gets full before his/her meal is finished	(1) ❑	(2) ❑	(3) ❑	(4) ❑	(5) ❑
My child enjoys eating	(1) ❑	(2) ❑	(3) ❑	(4) ❑	(5) ❑
My child eats more when she is happy	(1) ❑	(2) ❑	(3) ❑	(4) ❑	(5) ❑
My child is difficult to please with meals	(1) ❑	(2) ❑	(3) ❑	(4) ❑	(5) ❑
My child eats less when upset	(1) ❑	(2) ❑	(3) ❑	(4) ❑	(5) ❑
My child gets full up easily	(1) ❑	(2) ❑	(3) ❑	(4) ❑	(5) ❑
My child eats more when s/he has nothing else to do	(1) ❑	(2) ❑	(3) ❑	(4) ❑	(5) ❑

Please read the following statements and tick the boxes most appropriate to your child's eating behaviour

	Never	Rarely	Some-times	Often	Always
Even if my child is full up s/he finds room to eat his/her favourite food	(1) ❑	(2) ❑	(3) ❑	(4) ❑	(5) ❑
If given the chance, my child would drink continuously throughout the day	(1) ❑	(2) ❑	(3) ❑	(4) ❑	(5) ❑
My child cannot eat a meal if s/he has had a snack just before	(1) ❑	(2) ❑	(3) ❑	(4) ❑	(5) ❑
If given the chance, my child would always be having a drink	(1) ❑	(2) ❑	(3) ❑	(4) ❑	(5) ❑
My child is interested in tasting food s/he hasn't tasted before	(1) ❑	(2) ❑	(3) ❑	(4) ❑	(5) ❑
My child decides that s/he doesn't like a food, even	(1) ❑	(2) ❑	(3) ❑	(4) ❑	(5) ❑

	Never	Rarely	Some-times	Often	Always
without tasting it					
If given the chance, my child would always have food in his/her mouth	(1) ❑	(2) ❑	(3) ❑	(4) ❑	(5) ❑
My child eats more and more slowly during the course of a meal	(1) ❑	(2) ❑	(3) ❑	(4) ❑	(5) ❑

To which extent do you agree in the following statements?

	Strongly disagree	Disagree	Agree	Strongly agree
My child does not trust new foods	(1) ❑	(2) ❑	(3) ❑	(4) ❑
If my child doesn't know what's in a food, he or she won't try it	(1) ❑	(2) ❑	(3) ❑	(4) ❑
My child is afraid to eat things s/he has never had before	(1) ❑	(2) ❑	(3) ❑	(4) ❑
My child will eat almost anything	(1) ❑	(2) ❑	(3) ❑	(4) ❑
My child is very particular about the foods he or she will eat	(1) ❑	(2) ❑	(3) ❑	(4) ❑
My child is constantly sampling new and different foods	(1) ❑	(2) ❑	(3) ❑	(4) ❑

How often do you offer new and unknown food to the child before assuming the child doesn´t like the food?

(1) ❑ Once
(6) ❑ Twice
(5) ❑ 3-5 times
(2) ❑ 6-10 times
(3) ❑ 11 times or more

Here follow some questions about the child's appetite, eating behavior and how it usually is to feed the child. Think about how it has been the last month when answering the questions.

How agree or disagree are you in the following statements:

	Strongly disagree	Disagree	Neither-nor	Agree	Strongly agree
I have to be sure that my child does not eat too many sweets (candy, ice cream, cake, pastries)	(1) ❑	(2) ❑	(3) ❑	(4) ❑	(5) ❑
I have to be sure that my child does not eat too many high fat foods	(1) ❑	(2) ❑	(3) ❑	(4) ❑	(5) ❑
I have to be sure that my child does not eat too much of her favorite foods	(1) ❑	(2) ❑	(3) ❑	(4) ❑	(5) ❑
I intentionally keep some foods out of my child's reach	(1) ❑	(2) ❑	(3) ❑	(4) ❑	(5) ❑
I offer sweets (candy, ice cream, cake, pastries) to my child as a reward for good behavior	(1) ❑	(2) ❑	(3) ❑	(4) ❑	(5) ❑
I offer my child her favorite foods in exchange for good behavior	(1) ❑	(2) ❑	(3) ❑	(4) ❑	(5) ❑

How agree or disagree are you in the following statements:

	Strongly disagree	Disagree	Neither-nor	Agree	Strongly agree
If I did not guide or regulate my child's eating, she would eat too many junk foods	(1) ❑	(2) ❑	(3) ❑	(4) ❑	(5) ❑
If I did not guide or regulate my child's eating, she would eat too much of her favorite foods	(1) ❑	(2) ❑	(3) ❑	(4) ❑	(5) ❑
My child should always eat all of	(1) ❑	(2) ❑	(3) ❑	(4) ❑	(5) ❑

	Strongly disagree	Disagree	Neither-nor	Agree	Strongly agree
the food on her plate					
I have to be especially careful to make sure my child eats enough	(1) ❑	(2) ❑	(3) ❑	(4) ❑	(5) ❑
If my child says "I'm not hungry", I try to get her to eat anyway	(1) ❑	(2) ❑	(3) ❑	(4) ❑	(5) ❑
If I did not guide or regulate my child's eating, she would eat much less than she should	(1) ❑	(2) ❑	(3) ❑	(4) ❑	(5) ❑

And how often do you follow up ..

	Never	Rarely	Some-times	Often	Always
how much sweets (sweets, ice cream, cakes / biscuits etc.) your child eats	(1) ❑	(2) ❑	(3) ❑	(4) ❑	(5) ❑
how much snacks (potato chips, cheese pops and the like) your child eats?	(1) ❑	(2) ❑	(3) ❑	(4) ❑	(5) ❑
how much fatty food your child eats?	(1) ❑	(2) ❑	(3) ❑	(4) ❑	(5) ❑

Here are some questions regarding the child´s eating behavior and appetite and how you are dealing with feeding your child.There are no right or wrong answers to these questions, we are interested in what parents really feel and do.

Please read the following statements and tick the appropriate boxes to show how you deal with feeding your child.

	Never	Rarely	Sometimes	Often	Always
I allow my child to choose which foods to have for meal	(1) ❑	(2) ❑	(3) ❑	(4) ❑	(5) ❑
I give my child something to eat to make him/her feel better when s/he is feeling upset	(1) ❑	(2) ❑	(3) ❑	(4) ❑	(5) ❑

	Never	Rarely	Sometimes	Often	Always
I encourage my child to look forward to the meal	(1) ❑	(2) ❑	(3) ❑	(4) ❑	(5) ❑
I praise my child if s/he eats what I give him/her	(1) ❑	(2) ❑	(3) ❑	(4) ❑	(5) ❑
I decide how many snacks my child should have	(1) ❑	(2) ❑	(3) ❑	(4) ❑	(5) ❑
I encourage my child to eat a wide variety of foods	(1) ❑	(2) ❑	(3) ❑	(4) ❑	(5) ❑
In order to get my child to behave him/herself I promise him/her something to eat	(1) ❑	(2) ❑	(3) ❑	(4) ❑	(5) ❑
I present food in an attractive way to my child	(1) ❑	(2) ❑	(3) ❑	(4) ❑	(5) ❑
If my child misbehaves I withhold his/her favourite food	(1) ❑	(2) ❑	(3) ❑	(4) ❑	(5) ❑

Please read the following statements and tick the appropriate boxes to show how you deal with feeding your child

	Never	Rarely	Sometimes	Often	Always
I encourage my child to taste each of the foods I serve at mealtimes	(1) ❑	(2) ❑	(3) ❑	(4) ❑	(5) ❑
I allow my child to wander around during a meal	(1) ❑	(2) ❑	(3) ❑	(4) ❑	(5) ❑
I encourage my child to try foods that s/he hasn’t tasted before	(1) ❑	(2) ❑	(3) ❑	(4) ❑	(5) ❑
I give my child something to eat to make him/her feel better when s/he has been hurt	(1) ❑	(2) ❑	(3) ❑	(4) ❑	(5) ❑
I let my child decide when s/he would like to have her meal	(1) ❑	(2) ❑	(3) ❑	(4) ❑	(5) ❑

	Never	Rarely	Sometimes	Often	Always
I give my child something to eat if s/he is feeling bored	(1) ❑	(2) ❑	(3) ❑	(4) ❑	(5) ❑
I allow my child to decide when s/he has had enough snacks to eat	(1) ❑	(2) ❑	(3) ❑	(4) ❑	(5) ❑
I decide when it is time for my child to have a snack	(1) ❑	(2) ❑	(3) ❑	(4) ❑	(5) ❑
I use puddings as a bribe to get my child to eat his/her main course	(1) ❑	(2) ❑	(3) ❑	(4) ❑	(5) ❑

Please read the following statements and tick the appropriate boxes to show how you deal with feeding your child

	Never	Rarely	Sometimes	Often	Always
I encourage my child to enjoy his/her food	(1) ❑	(2) ❑	(3) ❑	(4) ❑	(5) ❑
I decide the times when my child eats his/her meals	(1) ❑	(2) ❑	(3) ❑	(4) ❑	(5) ❑
I give my child something to eat to make him/her feel better when s/he is worried	(1) ❑	(2) ❑	(3) ❑	(4) ❑	(5) ❑
I reward my child with something to eat when s/he is well behaved	(1) ❑	(2) ❑	(3) ❑	(4) ❑	(5) ❑
I let my child eat between meals whenever s/he wants	(1) ❑	(2) ❑	(3) ❑	(4) ❑	(5) ❑
I insist my child eats meals at the table	(1) ❑	(2) ❑	(3) ❑	(4) ❑	(5) ❑
I give my child something to eat to make him/her feel better when s/he is feeling angry	(1) ❑	(2) ❑	(3) ❑	(4) ❑	(5) ❑
I decide what my child eats between meals	(1) ❑	(2) ❑	(3) ❑	(4) ❑	(5) ❑

	Never	Rarely	Sometimes	Often	Always
I praise my child if s/he eats a new food	(1) ❑	(2) ❑	(3) ❑	(4) ❑	(5) ❑

Now follows the second part of the questionnaire with questions about yourself.
The questions deal with background information, eating habits and your experience of your own health.

What is your date of birth?
Must be written DD.MM:YYYY

_____

Have you become pregnant again after the child participating in the study was born?

(1) ☐ No
(2) ☐ Yes, I'm pregnant now
(3) ☐ Yes, I the child is born

Do you live with your child's other parent?
(1) ☐ Yes
(2) ☐ No

What civil status do you have now?

(1) ☐ Married
(2) ☐ Cohabitant
(3) ☐ Single
(4) ☐ Divorced
(5) ☐ Widow / widower
(6) ☐ Other, describe _____

What education do you have?
Choose the highest completed education
(1) ❑ Less than 9/10 years primary school
(2) ❑ Primary school
(3) ❑ High school
(4) ❑ Highschool, vocational

(5) ❑ University / college up to 4 years

(6) ❑ University / college more than 4 years

(8) ❑ Other education

What is your main activity now?

(1) ❑ Working full time

(2) ❑ Working part-time

(3) ❑ Homemakers

(4) ❑ Sick leave

(5) ❑ Leave

(6) ❑ Disability benefits

(7) ❑ During admission / rehabilitation

(8) ❑ Student

(9) ❑ Unemployed

(10) ❑ Other

Are you able to pay an unforeseen expence of 3000 NOK, e.g. for a repair or a dental bill?

(1) ❑ Yes

(2) ❑ No

(3) ❑ Don't know

Has it occurred during the past six months that you / you have had difficulties coping with expenses for food, transport, rent and the like?

(1) ❑ No, never

(2) ❑ Yes, seldom

(3) ❑ Yes, sometimes

(4) ❑ Yes, often

Now follow some questions about living habits and lifestyle:

How much do you weigh now?
Answer in kg _____

How tall are you? Answer in cm cm

_____

Do you smoke?

(1) ❑ No, never smoked regularly
(2) ❑ No, has quitted
(3) ❑ Yes, but not daily
(4) ❑ Yes, daily

Do you use snus (powdered tobacco)?

(1) ❑ No, never used snus regularly
(2) ❑ No, has quitted
(3) ❑ Yes, but not daily
(4) ❑ Yes, daily

Do you have the main responsibility for cooking at home?

(1) ❑ Yes
(2) ❑ No
(3) ❑ The responsibility is shared

How often do you do the following?

	Never	<1 / w	1 / w	2 / w	3 / w	4 / w	5 / w	6 / w	Daily
Cuts up vegetables	(1) ❑	(2) ❑	(3) ❑	(4) ❑	(5) ❑	(6) ❑	(7) ❑	(8) ❑	(9) ❑
Cuts up fruit	(1) ❑	(2) ❑	(3) ❑	(4) ❑	(5) ❑	(6) ❑	(7) ❑	(8) ❑	(9) ❑
Preparing dinner with raw materials	(1) ❑	(2) ❑	(3) ❑	(4) ❑	(5) ❑	(6) ❑	(7) ❑	(8) ❑	(9) ❑
Bake bread or rolls									

Then you have completed filling out the form.
You submit it by pressing the button below.

Thank you!
