# Supplementary material for: Examining the effects of an eHealth intervention from infant age 6 to 12 months on child eating behaviors and maternal feeding practices one year after cessation: The Norwegian randomized controlled trial Early Food for Future Health
Source: PLoS One. 2019 Aug 23;14(8):e0220437. doi: 10.1371/journal.pone.0220437 (PMC6707582; doi:10.1371/journal.pone.0220437)
Supplement: S3 File — (PDF) [file pone.0220437.s005.pdf]

REK
REGIONALE KOMITEER FOR MEDISINSK OG HELSEFAGLIG FORSKNINGSETIKK

Region:	Saksbehandler:	Telefon:	Vår dato:	Vår referanse:
REK sør-øst	Hege Holde Andersson	22845514	01.07.2015	2015/948

REK sør-øst B

Deres dato:	Deres referanse:
12.05.2015	

Vår referanse må oppgis ved alle henvendelser

Nina Cecilie Øverby
Universitetet i Agder

2015/948 Tidlig mat for fremtidig helse

Vi viser til søknad om forhåndsgodkjenning av ovennevnte forskningsprosjekt. Søknaden ble behandlet av Regional komité for medisinsk og helsefaglig forskningsetikk (REK sør-øst) i møtet 08.06.2015. Vurderingen er gjort med hjemmel i helseforskningsloven § 10, jf. forskningsetikkloven § 4.

Forskningsansvarlig: Universitetet i Agder **Prosjektleder:** Nina Cecilie Øverby

Prosjektleders prosjektbeskrivelse
«Hovedmålet med dette prosjektet er å utvikle, implementere og evaluere effekten av en e-helse intervensjon, som skal fremme et sunt og bærekraftig kosthold til spedbarn gjennom å oppmuntre foreldre til god matingspraksis. Prosjektet er en klynge-randomisert studie, hvor deltagere rekrutteres fra helsestasjonene før 6 måneders alder. Deltagere i kontroll og intervensjonsgruppen svarer på spørreskjema om kost og helse ved 6, 12, 24 og 48 måneders alder. Intervensjonen er en e-læringskomponent som styrker foreldrenes kunnskap og ferdigheter ved at de får se korte og motiverende filmer om hva som er viktig for den aktuelle alderen (6-12 måneder) barnet er i når det gjelder kosthold og samspill mellom foreldre og barn. Dagens foreldre er e-brukere, og forskning på hvordan e-formidling virker på barnets helse er mangelfull, dette prosjektet vil gi ny kunnskap om en enkel formidling av matingspraksis vil føre til bedre spisevaner og samspill med barnet med mulig betydning for senere helse.»

Komiteens vurdering
Formålet med prosjektet er å utvikle, implementere og evaluere effekten av en e-helse intervensjon, som skal fremme et sunt og bærekraftig kosthold til spedbarn gjennom å oppmuntre foreldre til god matingspraksis.

Helseforskningsloven gjelder for medisinsk og helsefaglig forskning, det vil si «virksomhet som utføres med vitenskapelig metodikk for å skaffe til veie ny kunnskap om helse og sykdom», jf. helseforskningsloven § 2, jf. § 4. Komiteen anser dermed at prosjektet ikke omfattes av helseforskningslovens virkeområde. Det kreves ingen forhåndsgodkjenning fra REK for å gjennomføre prosjektet.

Vedtak
Etter søknaden fremstår prosjektet ikke som medisinsk eller helsefaglig forskning, og det faller derfor utenfor helseforskningslovens virkeområde, jf. § 2.

Klageadgang
Du kan klage på komiteens vedtak, jf. forvaltningslovens § 28 flg. Klagen sendes til REK sør-øst B. Klagefristen er tre uker fra du mottar dette brevet. Dersom vedtaket opprettholdes av REK sør-øst B, sendes

klagen videre til Den nasjonale forskningsetiske komité for medisin og helsefag for endelig vurdering.

Komiteens avgjørelse var enstemmig.

Med vennlig hilsen

Geir Olav Hjortland nestleder REK sør-øst
B

Hege Holde Andersson
komitésekretær

Kopi til:
- *Dekan Stephen Seiler Universitetet i Agder*
- *Universitetet i Agder ved øverste administrative ledelse*

Region:	Saksbehandler:	Telefon:	Vår dato:	Vår referanse:
REK sør-øst	Hege Holde Andersson	22845514	01.07.2015	2015/948

REK sør-øst B

Deres dato:	Deres referanse:
12.05.2015	

Vår referanse må oppgis ved alle henvendelser

Nina Cecilie Øverby
Universitetet i Agder

2015/948 Early food for future health

We refer to the application for pre-approval of the above-mentioned research project. The application was considered by the Regional Committee for Medical and Health Research Ethics (REK south-east) at the meeting on 08.06.2015. The assessment is made on the basis of §10 of the Health Research Act, cf. § 4 of the Research Ethics Act.

Research director: University of Agder
Project manager: Nina Cecilie Øverby

Project manager's project description

«The purpose of this project is to develop, implement and evaluate the effects of an e-health intervention, aiming to promote a healthy and sustainable diet for infants by encouraging beneficial feeding practices. The project is a cluster-randomized study, in which participants are recruited from the municipalities´child health clinics before the age of 6 months. Participants in the control and intervention group will answer a questionnaire regarding diet and health at 6, 12, 24 and 48 months of age. The intervention is an e-learning component that aims to strengthen the parents' knowledge and skills by showing short and motivational films about what is important for the current age group (6-12 months) in terms of diet and interplay between parent and child. Today's parents are Internet users, and research on how e-communication may promote health is deficient. This project will provide new knowledge on whether e-communication of feeding practices will lead to better eating habits and possible significance for later health..»

The Committee's assessment

The purpose of the project is to develop, implement and evaluate the effects of an e-health intervention, which will promote a healthy and sustainable diet for infants by encouraging parents to have good feeding practices.
The Health Research Act applies to medical and health research, that is, "activities carried out with scientific methodology to provide new knowledge about health and illness", cf. § 2 of the Health Research Act, cf. the scope of the Health Research Act. No approval from REK is required to complete the project.

Decisions

According to the application, the project does not appear as medical or health research, and it therefore falls outside the scope of the Health Research Act., cf. § 2.

Besøksadresse:
Gullhaugveien 1-3, 0484 Oslo
Telefon: 22845511
E-post: post@helseforskning.etikkom.no
Web: http://helseforskning.etikkom.no/
All post og e-post som inngår i saksbehandlingen, bes adressert til REK sør-øst og ikke til enkelte personer
Kindly address all mail and e-mails to the Regional Ethics Committee, REK sør-øst, not to individual staff

Appeals
You may complain about the committee's decision, cf. § 28 of the Public Administration Act. The complaint is sent to REK south-east B. The time limit for appeal is three weeks from the time you receive this letter. If the decision is maintained by REK south-east B, the complaint is sent to the National Research Ethics Committee for Medicine and Health Sciences for final assessment.

The committee's decision was unanimous.

With best regards

Geir Olav Hjortland
Deputy REK south-east B

Hege Holde Andersson
Committee secretary

Copy to:
- *Dean Stephen Seiler University of Agder*
- *University of Agder at senior administrative management*
