# Supplementary material for: Examining the effects of an eHealth intervention from infant age 6 to 12 months on child eating behaviors and maternal feeding practices one year after cessation: The Norwegian randomized controlled trial Early Food for Future Health
Source: PLoS One. 2019 Aug 23;14(8):e0220437. doi: 10.1371/journal.pone.0220437 (PMC6707582; doi:10.1371/journal.pone.0220437)
Supplement: S4 File — (PDF) [file pone.0220437.s006.pdf]

# MELDESKJEMA

Meldeskjema (versjon 1.4) for forsknings- og studentprosjekt som medfører meldeplikt eller konsesjonsplikt (jf. personopplysningsloven og helseregisterloven med forskrifter).

Norsk samfunnsvitenskapelig datatjeneste AS
NORWEGIAN SOCIAL SCIENCE DATA SERVICES

## 1. Intro

| | | |
|---|---|---|
| Samles det inn direkte personidentifiserende opplysninger? | Ja ● Nei ○ | En person vil være direkte identifiserbar via navn, personnummer, eller andre personentydige kjennetegn.<br><br>Les mer om hva personopplysninger.<br><br>NB! Selv om opplysningene skal anonymiseres i oppgave/rapport, må det krysses av dersom det skal innhentes/registreres personidentifiserende opplysninger i forbindelse med prosjektet. |
| Hvis ja, hvilke? | ■ Navn<br>□ 11-sifret fødselsnummer<br>■ Adresse<br>■ E-post<br>■ Telefonnummer<br>■ Annet | |
| Annet, spesifiser hvilke | fødselsdato; dd.mm.aaaa. | |
| Skal direkte personidentifiserende opplysninger kobles til datamaterialet (koblingsnøkkel)? | Ja ● Nei ○ | Merk at meldeplikten utløses selv om du ikke får tilgang til koblingsnøkkel, slik fremgangsmåten ofte er når man benytter en databehandler |
| Samles det inn bakgrunnsopplysninger som kan identifisere enkeltpersoner (indirekte personidentifiserende opplysninger)? | Ja ○ Nei ● | En person vil være indirekte identifiserbar dersom det er mulig å identifisere vedkommende gjennom bakgrunnsopplysninger som for eksempel bostedskommune eller arbeidsplass/skole kombinert med opplysninger som alder, kjønn, yrke, diagnose, etc. |
| Hvis ja, hvilke | | |
| Skal det registreres personopplysninger (direkte/indirekte/via IP-/epost adresse, etc) ved hjelp av nettbaserte spørreskjema? | Ja ● Nei ○ | Les mer om nettbaserte spørreskjema. |
| Blir det registrert personopplysninger på digitale lyd-/bilde- eller videoopptak? | Ja ○ Nei ● | Bilde/videoopptak av ansikter vil regnes som personidentifiserende. For at stemme skal regnes som personidentifiserende, må denne bli registrert i kombinasjon med andre opplysninger, slik at personer kan gjenkjennes. |
| Søkes det vurdering fra REK om hvorvidt prosjektet er omfattet av helseforskningsloven? | Ja ● Nei ○ | NB! Dersom REK (Regional Komité for medisinsk og helsefaglig forskningsetikk) har vurdert prosjektet som helseforskning, er det ikke nødvendig å sende inn meldeskjema til personvernombudet (NB! Gjelder ikke prosjekter som skal benytte data fra pseudonyme helseregistre).<br><br>Dersom tilbakemelding fra REK ikke foreligger, anbefaler vi at du avventer videre utfylling til svar fra REK foreligger. |

## 2. Prosjekttittel

| | | |
|---|---|---|
| Prosjektittel | Early Food for Future Health<br>A randomised controlled trial evaluating the effect of an e-health intervention (BarnE-mat) in parents aiming to promote healthy and sustainable food habits from early childhood. | Oppgi prosjektets tittel. NB! Dette kan ikke være «Masteroppgave» eller liknende, navnet må beskrive prosjektets innhold. |

## 3. Behandlingsansvarlig institusjon

| | | |
|---|---|---|
| Institusjon | Universitetet i Agder | Velg den institusjonen du er tilknyttet. Alle nivå må oppgis. Ved studentprosjekt er det studentens tilknytning som er avgjørende. Dersom institusjonen ikke finnes på listen, har den ikke avtale med NSD som personvernombud. Vennligst ta kontakt med institusjonen. |
| Avdeling/Fakultet | Fakultet for helse- og idrettsvitenskap | |
| Institutt | Institutt for folkehelse, idrett og ernæring | |

## 4. Daglig ansvarlig (forsker, veileder, stipendiat)

| | | |
|---|---|---|
| Fornavn | Christine | Før opp navnet på den som har det daglige ansvaret for prosjektet. Veileder er vanligvis daglig ansvarlig |

| | | |
|---|---|---|
| Etternavn | Helle | ved studentprosjekt. |
| Stilling | Stipendiat | Veileder og student må være tilknyttet samme institusjon. Dersom studenten har ekstern veileder, kanbiveileder eller fagansvarlig ved studiestedet stå som daglig ansvarlig. |
| Telefon | 38142107 | |
| Mobil | 93069380 | Arbeidssted må være tilknyttet behandlingsansvarlig institusjon, f.eks. underavdeling, institutt etc. |
| E-post | christine.helle@uia.no | |
| Alternativ e-post | christine.helle2@gmail.com | NB! Det er viktig at du oppgir en e-postadresse som brukes aktivt. Vennligst gi oss beskjed dersom den endres. |
| Arbeidssted | Institutt for folkehelse, idrett og ernæring | |
| Adresse (arb.) | Postboks 422 | |
| Postnr./sted (arb.sted) | 4604 KRISTIANSAND | |
| Sted (arb.sted) | KRISTIANSAND | |

## 5. Student (master, bachelor)

| | | |
|---|---|---|
| Studentprosjekt | Ja ○ Nei ● | Dersom det er flere studenter som samarbeider om et prosjekt, skal det velges en kontaktperson som føres opp her. Øvrige studenter kan føres opp under pkt 10. |

## 6. Formålet med prosjektet

| | | |
|---|---|---|
| Formål | Formålet er å utvikle, implementere og evaluere effekten av en e-helse intervensjon som skal fremme et sunt og bærekraftig kosthold til spedbarn. Intervensjonen består av en nettside og syv månedlige filmer. Ved å gi lett tilgjengelig informasjon tilpasset barnets alder, ønsker en å styrke foreldres kunnskap og ferdigheter om barnets kosthold og samspillet mellom barn og forelder. Prosjektet er en klyngerandomisert studie, foreldre rekrutteres fra helsestasjonene når barnet er ca 5 mnd. Deltagere i ktr.- og interv.gruppe svarer på spørreskjema om kost og helse ved 6, 12, 24 og 48 mnd. alder. Prosjektet vil gi ny kunnskap om effekten av e-formidling som helsefremmende tiltak, og om dette fører til bedre spisevaner og samspill i målgruppen. | Redegjør kort for prosjektets formål, problemstilling, forskningsspørsmål e.l. |

## 7. Hvilke personer skal det innhentes personopplysninger om (utvalg)?

| | | |
|---|---|---|
| Kryss av for utvalg | □ Barnehagebarn<br>□ Skoleelever<br>□ Pasienter<br>■ Brukere/klienter/kunder<br>□ Ansatte<br>□ Barnevernsbarn<br>□ Lærere<br>□ Helsepersonell<br>□ Asylsøkere<br>■ Andre | |
| Beskriv utvalg/deltakere | Barn som er fra 5-14 mnd. gamle og deres foreldre. Rekrutteres fra helsestasjon. | Med utvalg menes dem som deltar i undersøkelsen eller dem det innhentes opplysninger om. |
| Rekruttering/trekking | Klynge-randomisert studie der helsestasjoner fordeles til kontroll- eller intervensjons-helsestasjon. Foreldre/barn rekrutteres fra helsetsasjonen til hhv. kontrollgruppe eller intervensjonsgruppe avhengig av hvilken helsestasjon de er knyttet til. | Beskriv hvordan utvalget trekkes eller rekrutteres og oppgi hvem som foretar den. Et utvalg kan trekkes fra registre som f.eks. Folkeregisteret, SSB-registre, pasientregistre, eller det kan rekrutteres gjennom f.eks. en bedrift, skole, idrettsmiljø eller eget nettverk. |
| Førstegangskontakt | Helsestasjonene kontaktes og rekrutteres av stipendiat i prosjektet.<br>Helsesøstre på helsestasjonen rekrutterer foreldre/barn til hhv. kontroll- eller intervensjonsgruppe. | Beskriv hvordan kontakt med utvalget blir opprettet og av hvem.<br>Les mer om dette på temasidene. |
| Alder på utvalget | ■ Barn (0-15 år)<br>□ Ungdom (16-17 år)<br>■ Voksne (over 18 år) | Les om forskning som involverer barn på våre nettsider. |

| | | |
|---|---|---|
| Omtrentlig antall personer som inngår i utvalget | 1000 stk, dvs. 500 foreldre/barn-dyader i hver gruppe. | |
| Samles det inn sensitive personopplysninger? | Ja ○ Nei ● | Les mer om sensitive opplysninger. |

| | | |
|---|---|---|
| Hvis ja, hvilke? | □ Rasemessig eller etnisk bakgrunn, eller politisk, filosofisk eller religiøs oppfatning<br>□ At en person har vært mistenkt, siktet, tiltalt eller dømt for en straffbar handling<br>□ Helseforhold<br>□ Seksuelle forhold<br>□ Medlemskap i fagforeninger | |
| Inkluderes det myndige personer med redusert eller manglende samtykkekompetanse? | Ja ○ Nei ● | Les mer om pasienter, brukere og personer med redusert eller manglende samtykkekompetanse. |
| Samles det inn personopplysninger om personer som selv ikke deltar (tredjepersoner)? | Ja ○ Nei ● | Med opplysninger om tredjeperson menes opplysninger som kan spores tilbake til personer som ikke inngår i utvalget. Eksempler på tredjeperson er kollega, elev, klient, familiemedlem. |
| **8. Metode for innsamling av personopplysninger** | | |
| Kryss av for hvilke datainnsamlingsmetoder og datakilder som vil benyttes | □ Papirbasert spørreskjema<br>■ Elektronisk spørreskjema<br>□ Personlig intervju<br>□ Gruppeintervju<br>□ Observasjon<br>□ Deltakende observasjon<br>□ Blogg/sosiale medier/internett<br>□ Psykologiske/pedagogiske tester<br>□ Medisinske undersøkelser/tester<br>□ Journaldata | Personopplysninger kan innhentes direkte fra den registrerte f.eks. gjennom spørreskjema,intervju, tester, og/eller ulike journaler (f.eks. elevmapper, NAV, PPT, sykehus) og/eller registre (f.eks.Statistisk sentralbyrå, sentrale helseregistre).<br><br>NB! Dersom personopplysninger innhentes fra forskjellige personer (utvalg) og med forskjellige metoder, må dette spesifiseres i kommentar-boksen. Husk også å legge ved relevante vedlegg til alle utvalgs-gruppene og metodene som skal benyttes. |
| | □ Registerdata | |
| | □ Annen innsamlingsmetode | |
| Tilleggsopplysninger | foreldre i begge grupper bes om å fylle ut et spørreskjema (ca 30 min) ved to anledninger; når barnet er ca 6 mnd. og når barnet er ca 12. mnd. Det spørres om bakgrunnsinformasjon samt kosthold/-vaner hos både barn og forelder. | |
| **9. Informasjon og samtykke** | | |
| Oppgi hvordan utvalget informeres | ■ Skriftlig<br>□ Muntlig<br>□ Informeres ikke | Vennligst send inn informasjonsskrivet eller mal for muntlig informasjon sammen med meldeskjema.<br><br>NB! Vedlegg lastes opp til sist i meldeskjemaet, se punkt 15 Vedlegg.<br><br>Last ned vår veiledende mal til informasjonsskriv.<br><br>Dersom utvalget ikke skal informeres om behandlingen av personopplysninger må det begrunnes. |
| Innhentes det samtykke fra utvalget? | ● Ja<br>○ Nei<br>○ Flere utvalg, ikke samtykke fra alle | Dersom det ikke skal innhentes samtykke, må det begrunnes. |
| Innhentes det samtykke fra foreldre for barn under 15 år? | Ja ● Nei ○ | Les mer om forskning som involverer barn og samtykke fra unge. |
| Hvis nei, begrunn | | |
| **10. Informasjonssikkerhet** | | |

| | | |
|---|---|---|
| Hvordan oppbevares navnelisten/ koblingsnøkkelen og hvem har tilgang til den? | Koblingsnøkkelen oppbevares på PC som er passordbeskyttet. Det er kun stipendiat og to veiledere som har tilgang til koblingsnøkkelen. | |
| Oppbevares direkte personidentifiserbare opplysninger på andre måter? | Ja ○ Nei ● | |
| Spesifiser | | NB! Som hovedregel bør ikke direkte personidentifiserende opplysninger registreres sammen med det øvrige datamaterialet. |

| | | |
|---|---|---|
| Hvordan registreres og oppbevares datamaterialet? | □ På server i virksomhetens nettverk<br>□ Fysisk isolert PC tilhørende virksomheten (dvs. ingen tilknytning til andre datamaskiner eller nettverk, interne eller eksterne)<br>■ Datamaskin i nettverkssystem tilknyttet Internett tilhørende virksomheten □ Privat datamaskin<br>□ Videoopptak/fotografi<br>□ Lydopptak<br>□ Notater/papir<br>□ Mobile lagringsenheter (bærbar datamaskin, minnepenn, minnekort, cd, ekstern harddisk, mobiltelefon)<br>□ Annen registreringsmetode | Merk av for hvilke hjelpemidler som benyttes for registrering og analyse av opplysninger.<br><br>Sett flere kryss dersom opplysningene registreres på flere måter.<br><br>Med «virksomhet» menes her behandlingsansvarlig institusjon.<br><br>NB! Som hovedregel bør data som inneholder personopplysninger lagres på behandlingsansvarlig sin forskningsserver.<br><br>Lagring på andre medier - som privat pc, mobiltelefon, minnepinne, server på annet arbeidssted - er mindre sikkert, og må derfor begrunnes. Slik lagring må avklares med behandlingsansvarlig institusjon, og personopplysningene bør krypteres. |
| Annen registreringsmetode beskriv | | |
| Hvordan er datamaterialet beskyttet mot at uvedkommende får innsyn? | Datamaskin beskyttet med brukernavn og passord i et låsbart rom. Ev. utskrifter oppbevares i låsbart arkivskap. | Er f.eks. datamaskintilgangen beskyttet med brukernavn og passord, står datamaskinen i et låsbart rom, og hvordan sikres bærbare enheter, utskrifter og opptak? |
| Samles opplysningene inn/behandles av en databehandler? | Ja ● Nei ○ | Dersom det benyttes eksterne til helt eller delvis å behandle personopplysninger, f.eks. Questback, transkriberingsassistent eller tolk, er dette å betrakte som en databehandler. Slike oppdrag må kontraktsreguleres. |
| Hvis ja, hvilken | SurveyXact / Rambøll | |
| Overføres personopplysninger ved hjelp av e-post/Internett? | Ja ○ Nei ● | F.eks. ved overføring av data til samarbeidspartner, databehandler mm.<br><br>Dersom personopplysninger skal sendes via internett, bør de krypteres tilstrekkelig. |
| Hvis ja, beskriv? | | |
| Skal andre personer enn daglig ansvarlig/student ha tilgang til datamaterialet med personopplysninger? | Ja ○ Nei ● | |
| Hvis ja, hvem (oppgi navn og arbeidssted)? | | |
| Utleveres/deles personopplysninger med andre institusjoner eller land? | ● Nei<br>○ Andre institusjoner<br>○ Institusjoner i andre land | F.eks. ved nasjonale samarbeidsprosjekter der personopplysninger utveksles eller ved internasjonale samarbeidsprosjekter der personopplysninger utveksles. |
| **11. Vurdering/godkjenning fra andre instanser** | | |
| Søkes det om dispensasjon fra taushetsplikten for å få tilgang til data? | Ja ○ Nei ● | For å få tilgang til taushetsbelagte opplysninger fra f.eks. NAV, PPT, sykehus, må det søkes om dispensasjon fra taushetsplikten. Dispensasjon søkes vanligvis fra aktuelt departement. |
| Hvis ja, hvilke | | |
| Søkes det godkjenning fra andre instanser? | Ja ● Nei ○ | F.eks. søke registereier om tilgang til data, en ledelse om tilgang til forskning i virksomhet, skole. |

| | | |
|---|---|---|
| Hvis ja, hvilken | Søknad ble sendt REK for godkjenning i mai. Søknaden er nå behandlet der. En fant at prosjektet ikke omfattes av helseforskningslovens virkeområde, og at det ikke kreves forhåndsgodkjenning fra REK for å gjennomføre prosjektet. | |

## 12. Periode for behandling av personopplysninger

| | | |
|---|---|---|
| Prosjektstart<br>Planlagt dato for prosjektslutt | 17.01.2016<br>31.01.2021 | Prosjektstart Vennligst oppgi tidspunktet for når kontakt med utvalget skal gjøres/datainnsamlingen starter.<br>Prosjektslutt: Vennligst oppgi tidspunktet for når datamaterialet enten skalanonymiseres/slettes, eller arkiveres i påvente av oppfølgingsstudier eller annet. |
| Skal personopplysninger publiseres (direkte eller indirekte)? | □ Ja, direkte (navn e.l.)<br>□ Ja, indirekte (bakgrunnsopplysninger)<br>■ Nei, publiseres anonymt | NB! Dersom personopplysninger skal publiseres, må det vanligvis innhentes eksplisitt samtykke til dette fra den<br>enkelte, og deltakere bør gis anledning til å lese gjennom og godkjenne sitater. |
| Hva skal skje med datamaterialet ved prosjektslutt? | ■ Datamaterialet anonymiseres<br>□ Datamaterialet oppbevares med personidentifikasjon | NB! Her menes datamaterialet, ikke publikasjon. Selv om data publiseres med personidentifikasjon skal som regel øvrig data anonymiseres.Med anonymisering menes at datamaterialet bearbeides slik at det ikke lenger er mulig å føre opplysningene tilbake til enkeltpersoner.<br>Les mer om anonymisering. |

## 13. Finansiering

| | | |
|---|---|---|
| Hvordan finansieres prosjektet? | Finansieres i sin helhet av Universitetet i Agder | |

## 14. Tilleggsopplysninger

| | | |
|---|---|---|
| Tilleggsopplysninger | Hovedveileder: Professor Nina C. Øverby, UiA<br>Biveileder: Elisabet R. Hillesund, UiA | |

# Reportform

Report form (version 1.4) for research and student projects that entail a duty of notification or a license obligation (cf. the Personal Data Act and the Health Register Act with regulations).

Norsk samfunnsvitenskapelig datatjeneste AS
NORWEGIAN SOCIAL SCIENCE DATA SERVICES

NSD

| 1. Introduction | | |
|---|---|---|
| **Which personal data will be processed?** | Yes ● No ○ | Personal data are any data about an identified or identifiable natural person (data subject). Pseudonymised data are also considered personal data. "Pseudonymisation" means processing collected data in way that the data can no longer be linked to individual persons, without the use of additional information. This usually involves removing identifiable information such as name, national ID number, contact details etc. from the collected data and giving each data subject a code/number. A scrambling key is the file/list of names and codes that makes it possible to identify individuals in the collected data. The scrambling key should be stored separately form the rest of the data. |
| If yes: | ■ Name<br>□ 11-digit national identification number<br>■ Adress<br>■ E-mail<br>■ Phone number<br>■ Other | |
| Other, specify: | Date of birth; dd.mm.yyyy. | |
| Will directly personal identification information be linked to the data material (by a scrambling key)? | Yes ● No ○ | NB: processing pseudonymised data is still considered processing personal data, even if you do not have access to the scrambling key, and even if the scrambling key is being stored by an external party, such as SSB, the National registry etc. |
| Will background information that can identify individuals (indirect personal identification information) be collected? | Yes ○ No ● | A person will be indirectly identifiable if it is possible to identify him / her through background information such as the municipality of residence or workplace / school combined with information such as age, gender, occupation, diagnosis, etc.. |
| Hvis ja, hvilke | | |
| Will personal data be registered (direct / indirect / via IP / email address, etc.) using web-based questionnaire? | Yes ● No ○ | Read more about online questionnaires: nettbaserte spørreskjema. |
| Will personal data be recorded on digital audio / video or video recordings? | Yes ○ No ● | Bilde/videoopptak av ansikter vil regnes som personidentifiserende. For at stemme skal regnes som personidentifiserende, må denne bli registrert i kombinasjon med andre opplysninger, slik at personer kan gjenkjennes. |
| Will there be sent an application to the Regional Committee for Medical and Health Research Ethics (REC) whether the project is covered by The Health Research Act? | Yes ● No ○ | NB! If REC has considered the project as health research, it is not necessary to submit a notification form to the NSD (NB! Does not apply to projects that will use data from pseudonymous health registers).<br>If feedback from REK is not yet available, we recommend that you await until the response from REK is available |
| **2. Project Information** | | |
| Title | Early Food for Future Health<br>A randomised controlled trial evaluating the effect of an e-health intervention (BarnE-mat) in parents aiming to promote healthy and sustainable food habits from early childhood. | The title must describe the content of the project |
| **3. Responsible Institution** | | |
| Institution<br>Department | University of Agder<br>Faculty of Health and Sport Sciences | Select the institution you are affiliated with. All levels must be stated. For student projects, it is the student's affiliation that applies. If the institution does not exist on the list, it does not have an agreement with NSD as a privacy officer. Please contact the institution. |
| **4. Daily manager (researcher, supervisor, research fellow)** | | |
| Firstname | Christine | List the name of the person who has the daily responsibility for the project. The supervisor is usually responsible |

| | | |
|---|---|---|
| Surname | Helle | In student projects. |
| Position | Stipendiat | Supervisor and student must be affiliated with the same institution. If the student has an external supervisor, the co- supervisor or the academic manager at the place of study, be responsible. |
| Phone | 38142107 | Workplace must be affiliated with treatment responsible institution, eg. subdivision, department etc. |
| Mobile phone | 93069380 | NB! It is important that you provide an active email address. Please let us know if it changes. |
| E-mail address | christine.helle@uia.no | |
| Alternative e-mail address | christine.helle2@gmail.com | |
| Workplace | Department of Public Health, Sport and Nutrition | |
| Address (work) | Postboks 422 | |
| | 4604 KRISTIANSAND | |
| City | KRISTIANSAND | |

## 5. Student (master, bachelor)

| | | |
|---|---|---|
| Student Project | Yes ○ No ● | If there are several students who collaborate on a project, a contact person will be chosen who will be listed here. Other students can be listed under item 10.. |

## 6. The purpose of the project

| | | |
|---|---|---|
| Purpose | The purpose is to develop, implement and evaluate the effect of an e-health intervention to promote a healthy diet for infants. The intervention consists of a website and seven monthly films. By providing easily accessible information adapted to the child's age, the intervention is aiming to strengthen the parents' knowledge and skills about infant nutrition and infant-parent interplay. The project is a cluster-randomized study, parents are recruited from the child health clinics when the child is about 5 months old. Participants will respond to questionnaires on diet and health at 6, 12, 24 and 48 months. age. The project will provide new knowledge about the use of Internet in health promoting, and whether this leads healthier child eating behaviors and more beneficial parental feeding practices in the intervention group. | Briefly explain the purpose of the project, issue, research questions, etc. |

## 7. From which persons will personal data be collected (sample)?

| | | |
|---|---|---|
| Kryss av for utvalg | □ Kindergarten children<br>□ School pupils<br>□ Patients<br>■ Users / clients / customers<br>□ Employees<br>□ Child welfare children<br>□ Teachers<br>□ Healthcare professionals<br>□ Asylum seekers<br>■ Other | |
| Describe the selection / participants | Children aged 5-14 months and their parents. Recruited from child health clinics. | By selection is meant those who participate in the survey or those for whom information is obtained. |
| Recruitment | Cluster-randomized study in which the child health clinics are distributed to either control or intervention. Parents / children are recruited from the child health clinics and randomized to control group or intervention group depending on the group status of the child health clinic they belong to. | Describe how the sample is recruited and state by whome. A selection can be drawn from registers such as. The Population Register, Statistics Norway registers, patient registers, or it can be recruited through eg. a business, school, sports environment or own network. |
| First Contact | The health centers are contacted and recruited by the study`s research fellow.<br>Health nurses at the child health clinic recruit parents / children to control or intervention group respectively. | Describe how contact with the sample is initiated and by whom.<br>Read more about this on the theme pages temasidene. |

| | | |
|---|---|---|
| Age of the sample | ■ Children (0-15 years)<br>□ Adolescence (16-17 years)<br>■ Adults (over 18 years) | Read about research that involves children / barn on our websites |
| Approximate number of persons included in the sample | 1000, ie 500 parent/infant -dyads in each group. | |
| Will sensitive personal information be collected? | Yes ○ No ● | Read more about sensitive information / sensitive opplysninger. |

| | | |
|---|---|---|
| If yes, which? | □ Racial or ethnic background, or political, philosophical or religious opinion<br>□ That a person has been suspected, charged or convicted of a criminal offense<br>□ Health conditions<br>□ Sexual Relationship<br>□ Trade union membership | |
| Will adults without or with reduced ability to consent be included? | Yes ○ No ● | Read more about patients, users and people with reduced or absent consent competence.<br>pasienter, brukere og personer med redusert eller manglende samtykkekompetanse. |
| Will personal information about people who do not participate (third parties) be collected | Yes ○ No ● | By third-party information is meant information that can be traced back to persons who are not included in the sample. Examples of third parties are colleague, student, client, family member. |
| **8. Method for collecting personal information** | | |
| Tic which data collection methods and data sources that will be used | □ Paper-based questionnaire<br>■ Electronic questionnaire<br>□ Personal interview<br>□ Group interview<br>□ Observation<br>□ Participating observation<br>□ Blog / social media / internet<br>□ Psychological / educational tests<br>□ Medical examinations / tests<br>□ Record data | Personal data can be obtained directly from the registered person eg. through questionnaires, interviews, tests, and / or various journals (eg student folders, NAV, PPT, hospital) and / or registers (eg Statistic Norway, central health records).<br><br>NB! If personal information is obtained from different persons (sample) and with different methods, this must be specified in the comment box. Also, remember to attach relevant attachments to all sample groups and methods to be used. |
| | □ Register data | |
| | □ Other collection method | |
| Additional information | parents in both groups are asked to fill out a questionnaire (about 30 min) on two occasions; when the child is about 6 months and when the child is about 12 months. It asks about background information and diet / habits in both children and the parent. | |
| **9. Information and consent** | | |
| State how the sample is informed | ■ Written<br>□ Oral<br>□ Not informed | Please submit the information letter or template for oral information together with the message form.<br><br>NB! Attachments are uploaded at the end of the message form, see section 15 Attachments<br><br>Download our tutorial template for the information letter - veiledende mal til informasjonsskriv.<br><br>If the sample is not to be informed about the processing of personal data, this must be justified. |
| Is consent obtained from the sample? | ● Yes<br>. No<br>○ Multiple samples not consent from everyone | If no consent is to be obtained, this must be justified. |
| Is there consent from parents for children under the age of 15? If no, justify | Yes ● No ○ | Read more about research that involves children and consent from adolescents |

| | | |
|---|---|---|
| | | |

## 10. Information Security

| | | |
|---|---|---|
| How is the name list / scrambling key stored and who has access to it? | A scrambling key is stored on a PC that is password protected. Only the research fellow and the two supervisors have access to the scrambling key. | |
| Will directly identifiable information be stored in other ways? | Yes ○ No ● | |
| Specify | | NB! As a rule, personal identifiable information should not be registered together with the other data material. |

| | | |
|---|---|---|
| How to register and store the data material | □ On server in the organization´s network<br>□ Physically isolated PC related business (ie no connection to other computers or networks, internal or external)<br>■ Computer in network system connected to the Internet of the organization<br>□ Private computer<br>□ Video recording / photography<br>□ Audio recording<br>□ Notes / Paper<br>□ Mobile Storage Devices (Laptop, Memory Stick, Memory Card, CD, External Hard Drive, Mobile Phone)<br>□ Other registration method | Tick for which tools that are used for registration and analysis of information.<br><br>Choose more crosses if the information is recorded in several ways.<br><br>By "organization" here is meant institution responsible for treatment.<br><br>NB! As a general rule, data containing personal data should be stored in the research leader's research server.<br><br>Storage on other media - such as a private PC, mobile phone, memory stick, server at another site - is less secure, and must therefore be justified. Such storage must be clarified with the institution responsible for treatment, and the personal data should be encrypted. |
| Other registration method, describe | | |
| How is the data material protected from unauthorized persons getting access? | Computer protected with username and password in a lockable room. Ev. prints are stored in lockable filing cabinets. | Is e.g. computer access protected by username and password, is the computer in a lockable room, and how are portable devices, prints and recordings secured? |
| Is the information collected or processed by a data processor? | Yes ● No ○ | If external people or organizations are used to completely or partially process personal data, eg. Questback, transcription assistant or interpreter, this is to be considered a data processor. Such assignments must be contract-regulated. |
| If so, which one | SurveyXact / Rambøll | |
| Is personal information transmitted using email / Internet? | Yes ○ No ● | Eg. when transferring data to a partner, data processor etc.<br>If personal information is to be sent via the internet, they should be sufficiently encrypted. |
| If so, describe? | | |
| Should people other than the daily responsible / student have access to the data material with personal data? | Yes ○ No ● | |
| If so, who (enter name and place of work)? | | |
| Will personal information be shared with other institutions or countries? | ● No<br>○ Other institutions<br>○ Institutions in other countries | Eg. by national collaborative projects where personal data is exchanged or by international collaborative projects where personal data is exchanged. |

## 11. Assessment / approval from other agencies

| | | |
|---|---|---|
| Will there be applied for an exemption from the duty of confidentiality in order to gain access to data? | Yes ○ No ● | In order to gain access to confidential information from e.g. NAV, PPT, hospital, you have to apply for exemption from the duty of confidentiality. Exemption is usually sought from the relevant ministry. |
| If so, which ones | | |

| | | |
|---|---|---|
| Are you seeking approval from other agencies? | Yes ● No ○ | Eg. apply for registries of access to data, a management about access to research in business, school. |
| If so, which ones | Application was sent to the Regional Committees for Medical and Health Research Ethics (REC) for approval in May. The application is now processed there. They concluded that the current project is not covered by the Health Research Act's scope, and that no prior approval from REK is required to carry out the project. | |
| **12. Period for processing personal data** | | |
| Project start<br>Scheduled date for project completion | 17.01.2016<br>31.01.2021 | Project start: Please state the time when the first contact with the sample is to be made / the data collection starts.<br>Project end: Please state the time when the data material is either to be anonymised / deleted, or archived pending follow-up studies or otherwise. |
| Should personal data be published (directly or indirectly)? | □ Yes, direct (name, etc.)<br>□ Yes, indirect (background information)<br>■ No, published anonymously | NB! If personal information is to be published, explicit consent must usually be obtained from individuals, and participants should be given the opportunity to read through and approve quotes. |
| What will happen to the data material at the end of the project? | ■ The data material is anonymised<br>□ The data material is stored with personal identification | NB! Here is meant the data material, not the publication. Although data is published with personal identification, other data is usually anonymised. By anonymisation is meant that the data material is processed so that it is no longer possible to connect the information to individuals.<br>Read more about anonymization / anonymisering. |
| **13. Finance** | | |

| | | |
|---|---|---|
| How is the project financed?? | Financed entirely by the University of Agder | |
| **14. Additional information** | | |
| Additional information | Main supervisor: Professor Nina C. Øverby, UiA<br>Supervisor: Elisabet R. Hillesund, UiA | |

**Norsk samfunnsvitenskapelig datatjeneste AS**
NORWEGIAN SOCIAL SCIENCE DATA SERVICES

Harald Hårfagres gate 29
N-5007 Bergen
Norway
Tel: +47-55 58 21 17
Fax: +47-55 58 96 50
nsd@nsd.uib.no
www.nsd.uib.no
Org.nr. 985 321 884

Christine Helle
Institutt for folkehelse, idrett og ernæring Universitetet i Agder
Serviceboks 422
4604 KRISTIANSAND S

Vår dato: 17.08.2015 Vår ref: 43975 / 3 / MSI Deres dato: Deres ref:

TILBAKEMELDING PÅ MELDING OM BEHANDLING AV PERSONOPPLYSNINGER

Vi viser til melding om behandling av personopplysninger, mottatt 03.07.2015. Meldingen gjelder prosjektet:

| | |
|---|---|
| *43975* | *Early Food for Future Health. A randomised controlled trial evaluating the effect of an e-health intervention (BarnE-mat) in parents aiming to promote healthy and sustainable food habits from early childhood* |
| *Behandlingsansvarlig* | *Universitetet i Agder, ved institusjonens øverste leder* |
| *Daglig ansvarlig* | *Christine Helle* |

Personvernombudet har vurdert prosjektet, og finner at behandlingen av personopplysninger vil være regulert av § 7-27 i personopplysningsforskriften. Personvernombudet tilrår at prosjektet gjennomføres.

Personvernombudets tilråding forutsetter at prosjektet gjennomføres i tråd med opplysningene gitt i meldeskjemaet, korrespondanse med ombudet, ombudets kommentarer samt personopplysningsloven og helseregisterloven med forskrifter. Behandlingen av personopplysninger kan settes i gang.

Det gjøres oppmerksom på at det skal gis ny melding dersom behandlingen endres i forhold til de opplysninger som ligger til grunn for personvernombudets vurdering. Endringsmeldinger gis via et eget skjema, http://www.nsd.uib.no/personvern/meldeplikt/skjema.html. Det skal også gis melding etter tre år dersom prosjektet fortsatt pågår. Meldinger skal skje skriftlig til ombudet.

Personvernombudet har lagt ut opplysninger om prosjektet i en offentlig database, http://pvo.nsd.no/prosjekt.

Personvernombudet vil ved prosjektets avslutning, 31.01.2021, rette en henvendelse angående status for behandlingen av personopplysninger.

Vennlig hilsen

Vigdis Namtvedt Kvalheim

Marte Byrkjeland

Kontaktperson: Marte Byrkjeland tlf: 55 58 33 48

*Dokumentet er elektronisk produsert og godkjent ved NSDs rutiner for elektronisk godkjenning.*

# Personvernombudet for forskning

## Prosjektvurdering - Kommentar

Prosjektnr: 43975

Prosjektleder oppgir at prosjektet er meldt til REK, hvor en fant at prosjektet ikke omfattes av helseforskningsloven. Personvernombudet ber om at kopi fra REK sendes til personvernombudet@nsd.uib.no for arkivering i saksmappe.

Formålet er å utvikle, implementere og evaluere effekten av en e-helse intervensjon som skal fremme et sunt og bærekraftig kosthold til spedbarn.

Utvalget informeres skriftlig om prosjektet og samtykker til deltakelse. Informasjonsskrivene er godt utformet, men setningen om at studien er godkjent av REK må slettes. Kontaktopplysninger til forsker/daglig ansvarlig bør oppgis.

Personvernombudet finner at det vil behandles sensitive personopplysninger om helseforhold.

Personvernombudet legger til grunn at forsker etterfølger Universitetet i Agder sine interne rutiner for datasikkerhet.

SurveyXact/Rambøll er databehandler for prosjektet. Universitetet i Agder skal inngå skriftlig avtale med SurveyXact/Rambøll om hvordan personopplysninger skal behandles, jf. personopplysningsloven § 15. For råd om hva databehandleravtalen bør inneholde, se Datatilsynets veileder: http://www.datatilsynet.no/Sikkerhetinternkontroll/Databehandleravtale/.

Forventet prosjektslutt er 31.01.2021. Ifølge prosjektmeldingen skal innsamlede opplysninger da anonymiseres. Anonymisering innebærer å bearbeide datamaterialet slik at ingen enkeltpersoner kan gjenkjennes. Det gjøres ved å:
- slette direkte personopplysninger (som navn/koblingsnøkkel)
- slette/omskrive indirekte personopplysninger (identifiserende sammenstilling av bakgrunnsopplysninger somf.eks. bosted/arbeidssted, alder og kjønn).

Vi gjør oppmerksom på at også databehandler (SurveyXact/Rambøll) må slette personopplysninger tilknyttet prosjektet i sine systemer. Dette inkluderer eventuelle logger og koblinger mellom IP-/epostadresser og besvarelser.

**Translation from Norwegian to English**:

## RESPONSE TO APPLICATION FOR TREATMENT OF PERSONAL INFORMATION

We refer to the application for processing of personal data, received on 03.07.2015. The application concerns the following project:

| | |
|---|---|
| 43975 | *Early Food for Future Health. A randomized controlled trial evaluating the effect of an e-health intervention (BarnE-mat) in parents aiming to promote healthy and sustainable food habits from early childhood* |
| Coordinator: | University of Agder, at the head of the department |
| Daily responsible: | Christine Helle |

The Norwegian Social Science Data Services (NSD) has considered the project, and finds that the processing of personal data will be regulated by section 7-27 of the Personal Information Regulations. NSD recommends that the project can be completed.

NSD`s recommendation implies that the project is conducted in accordance with the information given in the notification form, in correspondence with the NSD, the NSD's comments as well as according to the Personal Health Data Filing System Act with regulations. The processing of personal data can be started.

A notification should be given if the treatment changes in relation to the information on which NSD's assessment is based. A notification of change is given through a separate form; http://www.nsd.uib.no/personvern/meldeplikt/skjema.html. It shall also be sent a notification to NSD after three years if the project is still in progress. Notifications to NSD must be made in writing.

NSD has published information about the project in a public database, http://pvo.nsd.no/prosjekt.

By the end of the project, 31.01.2021, NSD will address a request for the current status of processing personal data.

Kindly Regards

Vigdis Namtvedt Kvalheim    Marte Byrkjeland

Contact person: Marte Byrkjeland
tel: 55 58 33 48

# The Data Protection Office for Research

## Project assessment - Comments

Project number: 43975

The project manager states that the project has been reported to Regional Ethic Committee (REK), where one found that the project is not covered by the Health Research Act. The Data Protection Office requests that a copy from REK be sent to personvernombudet@nsd.uib.no for filing in the case file.

The purpose is to develop, implement and evaluate the effect of an e-health intervention aiming to promote a healthy and sustainable diet for infants.

The study sample is informed about the project and agrees to participation. The information is well written, but the statement that the study is approved by REK must be deleted. Contact information for the researcher / daily responsible should be stated.

The Data Protection Office finds that sensitive personal information about health conditions will be processed.

The Data Protection Office assumes that the researcher follows the University of Agder's internal data security routines.

SurveyXact / Rambøll is the data processor for the project. The University of Agder will have a written agreement with SurveyXact / Rambøll on how personal data shall be processed, cf. § 15 of the Personal Data Act.

Expected project end is 31.01.2021. According to the study protocol, collected information should then be anonymised. Anonymization involves processing the data so that no individual can be recognized. This is done by:

- deleting direct personal information (as name / link key)

- deleting / rewriting indirect personal information (identifying compilation of background information such as residence / work place, age and gender).

We note that data processor (SurveyXact / Rambøll) must also delete personal data associated with the project in their systems. This includes any logs and links between IP / email addresses and responses.
