# Supplementary material for: Examining the effects of an eHealth intervention from infant age 6 to 12 months on child eating behaviors and maternal feeding practices one year after cessation: The Norwegian randomized controlled trial Early Food for Future Health
Source: PLoS One. 2019 Aug 23;14(8):e0220437. doi: 10.1371/journal.pone.0220437 (PMC6707582; doi:10.1371/journal.pone.0220437)
Supplement: S5 File — (PDF) [file pone.0220437.s007.pdf]

Endringsskjema

for endringer i forsknings- og studentprosjekt som medfører meldeplikt eller konsesjonsplikt
(jf. personopplysningsloven og helseregisterloven med forskrifter)

Endringsskjema sendes per e-post
personvernombudet@nsd.uib.no *til:*

1. PROSJEKT	
Navn på daglig ansvarlig: Christine Helle	Prosjektnummer:<br>43975
Evt. navn på student:	

2. BESKRIV ENDRING(ENE)	
Endring av daglig ansvarlig/veileder:	*Ved bytte av daglig ansvarlig må bekreftelse fra tidligere og ny daglig ansvarlig vedlegges. Dersom vedkommende har sluttet ved institusjonen, må bekreftelse fra representant på minimum instituttnivå vedlegges.*
Endring av dato for anonymisering av datamaterialet:	*Ved forlengelse på mer enn ett år utover det deltakerne er informert om, skal det fortrinnsvis gis ny informasjon til deltakerne.*
Gis det ny informasjon til utvalget? Ja: X Nei: ____ Hvis nei, begrunn:<br>Informasjon til utvalget endres kun i henhold til endret utvalg og rekrutteringsmåte, se under	
Endring av metode(r):	*Angi hvilke nye metoder som skal benyttes, f.eks. intervju, spørreskjema, observasjon, registerdata, osv.*
Endring av utvalg:<br>Påmelding til studien skjer via en åpen nettside der foreldre med barn i aktuell alder selv kan melde seg på. Dette er uendret fra tidligere. Informasjonsskriv til foreldre vil ligge på den åpne nettsiden.<br><br>Endring av rekruttering, utvalg og randomisering mtp. følgende:<br>Fra å gjelde tre fylker (Oppland, Rogaland og Aust-Agder) utvides det geografiske området til å innbefatte hele landet.<br>Informasjon om studien sendes per epost til alle landets helsestasjoner gjennom kommunenes postmottak (ikke-personlig epost). Kjøp av adresser gjennom kommuneforlaget. Avsender (Universitetet i Agder) skal komme tydelig frem. I eposten orienteres det om bakgrunn for studien, se vedlegg. Eposten vil også inneholde 2 pdf-filer (plakat og brosjyre) som den enkelte helsestasjon selv kan skrive ut. Deltagere kan rekrutteres ved å se på plakat/brosjyre, følge beskrivelse der og gå inn på nettsiden og melde seg på.<br>Deltagere rekrutteres i tillegg gjennom bruk av sosiale medier (Facebook). Det lages en egen «annonse» der det tydelig kommer frem at UiA er avsender. Annonsen inneholder lenke til studiens åpne hjemmeside.	*Dersom det er snakk om små endringer i antall deltakere er endringsmelding som regel ikke nødvendig. Ta kontakt på telefon før du sender inn skjema dersom du er i tvil.*
Annet:	

3. TILLEGGSOPPLYSNINGER

Har du spørsmål i forbindelse med utfylling av skjemaet, ta gjerne kontakt med Personvernombudet hos NSD, telefon 55 58 81 80

4. ANTALL VEDLEGG

Utkast informasjonsskriv helsestasjon<br>Utkast informasjonsskriv foreldre	*Legg ved eventuelle nye vedlegg (informasjonsskriv, intervjuguide, spørreskjema, tillatelser, og liknende.)*

Har du spørsmål i forbindelse med utfylling av skjemaet, ta gjerne kontakt med Personvernombudet hos NSD, telefon 55 58 81 80

Change notification

for changes in research and student projects that entail a duty of notification or a license obligation
(see the Personal Data Act and the Health Register Act with Regulations)

Change form is sent by e-mail to
personvernombudet@nsd.uib.no

1. PROJECT	
Name of daily manager: Christine Helle	Project numberr:<br>43975
Name of potential student:	

2. DESCRIPTION OF CHANGE (ONE)	
Change of daily manager / supervisor:	*When changing the responsible person, confirmation from the previous and new responsible person must be enclosed.*<br>*If he or she has ended their work at the institution, confirmation from a representative at the department level must be enclosed.*<br>.
Change of date for anonymization of the data material:	*If the extension lasts more than one year beyond what the participants are informed about, new information should preferably be given to the participants..*
Will the new information be given to the participants? Yes: X No: ____ If no, justify:<br>Information for the study sample is only changed according to geographical area and the changed recruitment method, see below	
Change of method (s):	*Specify which new methods are to be used, e.g. interview, questionnaire, observation, registry data, etc.*
Change of sample:<br>Registration for the study is done through an open website where parents with children in the relevant age group can register themselves. This is unchanged from previous. Information to parents will be posted on the open website.<br><br>Change of recruitment, selection and randomization:<br>From previously including three counties (Oppland, Rogaland and Aust-Agder), the geographical area is expanded to include the entire country (Norway).<br>Information about the study is sent by e-mail to all Norwegian child health clinics through the municipalities' mail reception system (non-personal e-mail). Purchase of addresses will be done through the municipality publisher. The sender (the University of Agder) must appear clearly. In the email, the background for the study is described, see appendix. The e-mail will also contain 2 pdf files (poster and brochure/hand out), which the individual child health clinic may print out. Participants can be recruited by receiving the brochure, follow the description there and go to the study´s website for registration.<br>Participants are also recruited through use of social media (Facebook). A separate online advertisement is made where it is clear that the UiA is the sender. The adverticement contains a link to the study's open website.	*If there are small changes in the number of participants, the change notification is usually not necessary. If in doubt, please contact by phone before submitting a form.*
Other:	

3. ADDITIONAL INFORMATION

4. NUMBER OF ATTACHMENTS

Draft information child health clinics<br>Draft information parents	*Attach any new attachments (information letter, interview guide, questionnaire, permissions, and the like.)*

If you have any questions in connection with completing the form, please contact the Data Protection Officer at NSD, telephone +45 55 58 81 80

Fre. 18.12.2015 11.31

Marte Byrkjeland marte.byrkjeland@nsd.no

Prosjektnr: 43975. Early Food for Future Health. A randomised controlled trial evaluating the effect of an e-health intervention (BarnE-mat) in parents aiming to promote healthy and sustainable food habits from early childhood

TILBAKEMELDING PÅ ENDRINGSMELDING

Vi viser til endringsmelding mottatt 20.11.2015 for prosjekt:

43975. Early Food for Future Health. A randomised controlled trial evaluating the effect of an e-health intervention (BarnE-mat) in parents aiming to promote healthy and sustainable food habits from early childhood

Endringen gjelder rekruttering og utvalgets størrelse. Personvernombudet har registrert endringene og har ingen spesielle kommentarer til disse.

Revidert informasjonsskriv til foreldrene er godt utformet, men i Kapittel B under "Utlevering av materiale og opplysninger til andre" må det gjøres noen små justeringer. At man gir sitt samtykke til utlevering av "prøver" må slettes, da dette ikke er aktuelt siden det ikke skal innhentes noen prøver i prosjektet, jf. "Undersøkelser". Videre bør formålet (forskningsformål) med eventuell utlevering til andre ved UiA presiseres.

Vi legger til grunn at prosjektopplegget for øvrig er uendret.
Ta gjerne kontakt om noe er uklart.

Vennlig hilsen / Best regards

Marte Byrkjeland
Rådgiver / Adviser

Norsk samfunnsvitenskapelig datatjeneste AS
(Norwegian Social Science Data Services)

Personvernombud for forskning
(Data Protection Official for Research)

Harald Hårfagres gate 29, 5007 BERGEN

Tlf. sentral: (+47) 55 58 81 80

Tlf. direkte: (+47) 55 58 36 01

Epost: marte.byrkjeland@nsd.no

www.nsd.uib.no/personvern

Projectnr 43975.
Decision NSD.msg

TRANSLATION OF EMAIL FROM NORWEGIAN SOCIAL SCIENCE DATA SERVICES / DATA PROTECTION OFFICIAL FOR RESEARCH CONCERNING CHANGE-NOTIFICATION

Friday 18.12.2015 11.31

Marte Byrkjeland marte.byrkjeland@nsd.no

Prosjektnr: 43975. Early Food for Future Health. A randomised controlled trial evaluating the effect of an e-health intervention (BarnE-mat) in parents aiming to promote healthy and sustainable food habits from early childhood

RECOMMENDATIONS CONCERNING CHANGE-NOTIFICATION

We refer to change-notification received on 20.11.2015 for project:

43975. Early Food for Future Health. A randomised controlled trial evaluating the effect of an e-health intervention (BarnE-mat) in parents aiming to promote healthy and sustainable food habits from early childhood

The change relates to recruitment and the size of the sample. NSD (Norwegian Social Science Data Services) has registered the changes and has no special comments on these.

The revised information letter for parents is well-designed, but in Chapter B under "Submission of material and information to others", some minor adjustments must be made. To give consent for the delivery of "samples" must be deleted, as this is not relevant since no samples will be obtained in the project. Furthermore, the purpose (research purpose) with possible disclosure to others at the University of Agder should be clarified.

We assume that the project plan is otherwise unchanged.

Feel free to contact us if something is unclear.

Best regards

Marte Byrkjeland
Adviser

Norwegian Social Science Data Services
Data Protection Official for Research
Harald Hårfagres gate 29, 5007 BERGEN
NORWAY

Tel. switchboard: (+47) 55 58 81 80

Direct number: (+47) 55 58 36 01

Email: marte.byrkjeland@nsd.no

www.nsd.uib.no/personvern
