# Supplementary material for: Examining the effects of an eHealth intervention from infant age 6 to 12 months on child eating behaviors and maternal feeding practices one year after cessation: The Norwegian randomized controlled trial Early Food for Future Health
Source: PLoS One. 2019 Aug 23;14(8):e0220437. doi: 10.1371/journal.pone.0220437 (PMC6707582; doi:10.1371/journal.pone.0220437)

S1 Figure:

Mean Weigh-for-age z-score and mean BMI z-score for the control and intervention groups from birth to 24 months of age including all available data

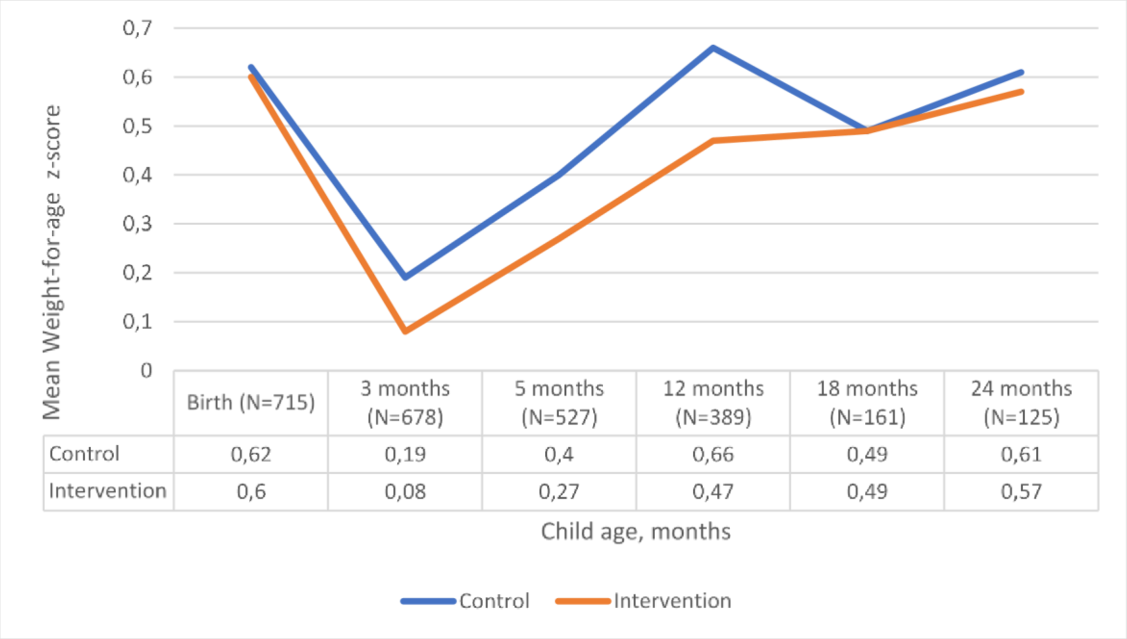

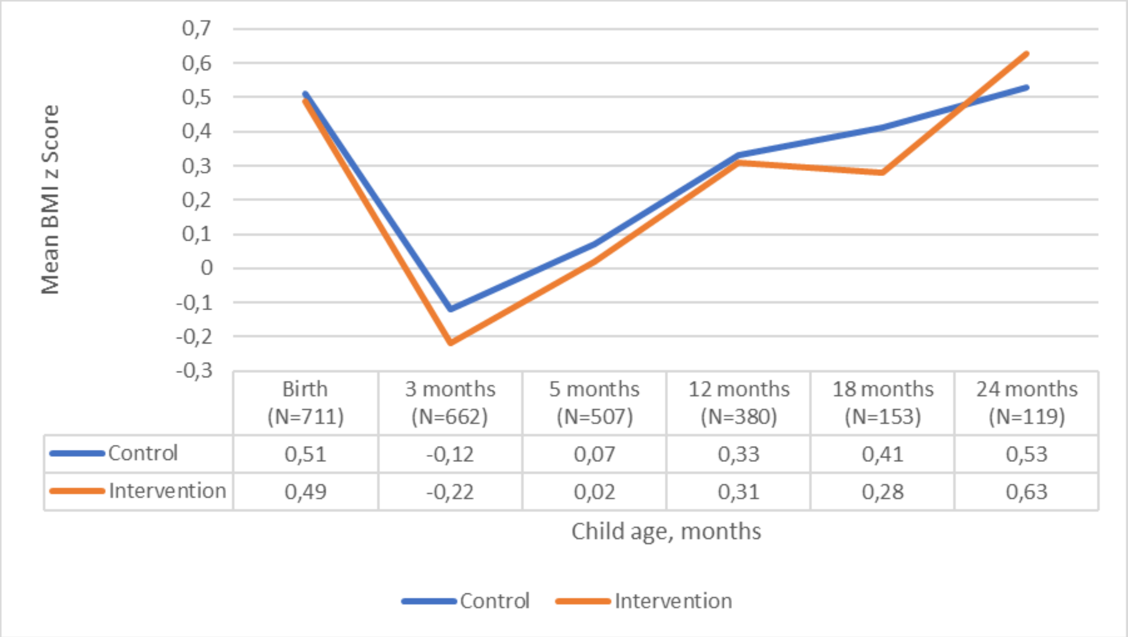

Supplement: S1 Fig — (PDF) [file pone.0220437.s008.pdf]
